# 2-(Trihydroxyphenyl)thienopyrimidinones as Key Scaffolds for Targeting a Novel Allosteric Site of HIV-1 Integrase

**DOI:** 10.3390/molecules30244709

**Published:** 2025-12-09

**Authors:** Graziella Tocco, Antonio Laus, Mattia Casula, Pierluigi Caboni, John A. Beutler, Enzo Tramontano, Francesca Esposito

**Affiliations:** 1 Department of Life and Environmental Sciences, University of Cagliari, Cittadella Universitaria di Monserrato, 09042 Monserrato, CA, Italy; laus@crs4.it (A.L.); mattia.casula@unica.it (M.C.); caboni@unica.it (P.C.); tramon@unica.it (E.T.); 2 Center for Advanced Studies Research and Development in Sardinia (CRS4), 09050 Pula, CA, Italy; 3 Molecular Targets Program, National Cancer Institute, Frederick, MD 21702, USA; beutlerj@mail.nih.gov

**Keywords:** HIV-1 Integrase, sucrose binding site, novel integrase allosteric inhibitors

## Abstract

A promising novel class of 2-(trihydroxyphenyl)thieno[2,3-*d*]pyrimidin-4(3*H*)-ones as HIV-1 integrase (IN) allosteric inhibitors is reported. All compounds were considerably more effective than the control compound LEDGIN-6, showing an inhibitory activity in the low micromolar range in both the HIV-1 integrase LEDGF/p75-dependent and independent activity assays. Selected compounds **6**, **9**, **10**, and **12** were also able to affect the exchange of the HIV-1 IN-IN subunit and the interaction between IN and LEDGF/p75. Differently from LEDGINs, small-molecule inhibitors were able to interact with the IN binding domain (IBD) of Lens-Epithelium-derived Growth Factor/p75 (LEDGF/p75) and HIV-1 IN, and none of these compounds was able to induce multimerization, suggesting a different mechanism of action. In silico experiments carried out on compounds **6** and **10** suggest that a region adjacent to the sucrose binding site (SBS), together with a second nearby cavity, may represent relevant allosteric regions for their interaction with IN. In vitro experiments performed in the presence and absence of sucrose on selected IN mutants are qualitatively consistent with this model, although further structural and biophysical studies will be required to define the exact binding mode.

## 1. Introduction

Integration of a retrotranscribed viral DNA (ssRNA) copy into the host genome is a critical step in the retroviral replication cycle. This process, catalysed by HIV-1 integrase (IN), is essential for viral replication and pathogenesis. The absence of a human homolog makes IN an attractive therapeutic target with potentially fewer side effects.

IN is a 32 kDa multimeric enzyme comprising three domains: the N-terminal domain (NTD) with a conserved zinc-binding motif for folding and multimerization [1,2,3]; the catalytic core domain (CCD) containing the D64-D116-E152 motif that coordinates magnesium ions for activity; and the C-terminal domain (CTD) involved in viral and host DNA binding. All domains are required for functional oligomerization.

Integration occurs in two steps: 3′-processing in the cytoplasm, where IN dimers bind viral DNA and remove a dinucleotide adjacent to the CA sequence, followed by strand transfer in the nucleus [4,5]. Here, two DNA-bound dimers form an active tetramer with LEDGF/p75, enabling insertion of viral DNA into the host genome [6].

Currently, only strand transfer inhibitors (INSTIs) are FDA-approved, but resistance to second-generation drugs such as Bictegravir [7] and Cabotegravir [8] underscores the need for alternative strategies. This has led to the development of allosteric IN inhibitors (ALLINIs/LEDGINs or NCINIs), which target non-catalytic sites to disrupt IN function [9,10].

At present, the predominant strategies for allosteric inhibition of IN primarily concentrate on modifying IN oligomerization by interfering with the assembly of oligomeric complexes [11,12,13] or encouraging abnormal IN multimerization [14,15,16,17]. Specifically, LEDGINs imitate the interaction between IN and LEDGF/p75 [18,19,20,21], thereby disrupting the protein–protein interactions critical for the process of viral replication, promoting IN aberrant multimerization, and inhibiting the HIV-1 IN strand-transfer catalytic activity both in the presence and absence of LEDGF/p75 protein. LEDGINs can allosterically regulate the dynamic interactions of IN subunits. This regulation not only encourages and stabilizes the multimerization of IN but also prevents the IN-IN subunit exchange essential for the IN-DNA complex formation [13].

Located at the dimer interface of the IN CCD, the LEDGF/p75 IBD is strategically positioned, enabling the potential for allosteric inhibition or modulation of IN activity by focusing on this specific site. In fact, several small molecules with different chemical properties have been recently developed to exploit this potential [22,23,24,25,26,27,28,29].

The allure of the CCD interface has significantly increased following the recent discovery of a new allosteric site, known as SBS. This site was serendipitously uncovered through the crystallization of a complex that included a sucrose molecule within the IN CCD [30]. Subsequent in silico studies indicated that the presence of sucrose not only enhances the binding free energy of the two monomers, thereby promoting dimer formation but can also boost the efficacy of the first-generation drug Raltegravir. Furthermore, studies also indicate that the binding of sucrose to the CCD interface improves the inhibitory effect of the LEDGIN derivative CX0516 via a synergistic mechanism that stabilizes the multimeric structure of IN, thereby impeding its catalytic activity [31].

**Figure 1:**
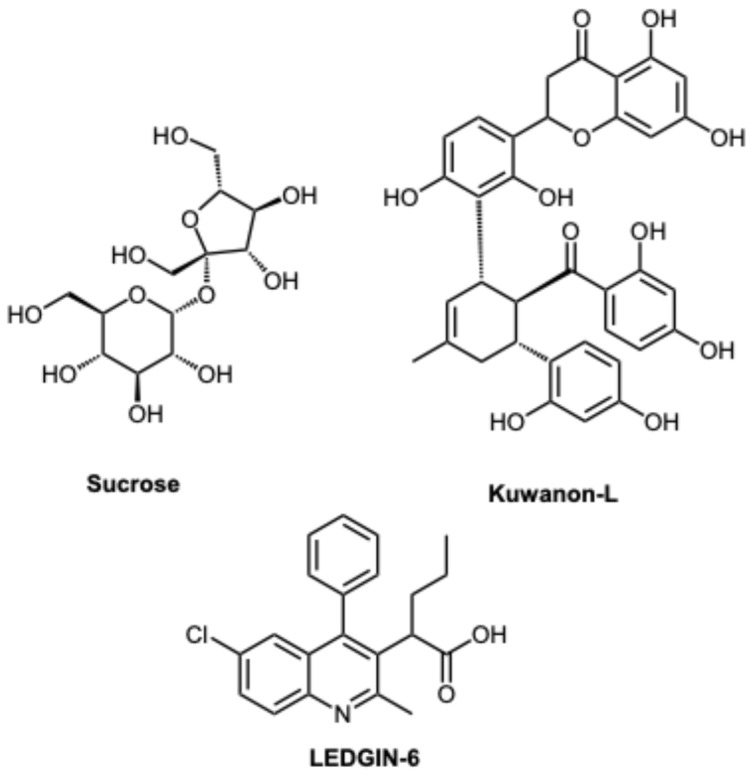
Chemical structures of sucrose, Kuwanon-L and LEDGIN-6.

Subsequently, a structure-based virtual screening approach identified the natural 3′-prenylated flavanone Kuwanon-L (Figure 1) as a molecule able to bind to the Sucrose Binding Site (SBS). In vitro assays corroborated the in silico findings, demonstrating the ability of Kuwanon-L to inhibit IN activity both in the absence and in the presence of LEDGF/p75, akin to CX0156 [32]. Further investigations also demonstrated the ability of Kuwanon-L to bind to HIV-1 Reverse Transcriptase (RT), affecting both Ribonuclease H (RNase H) and RNA-dependent DNA polymerase (RdRp) activities [33].

From a structural standpoint, the presence of polyphenolic units enables Kuwanon-L to engage with IN and RT effectively, as also evidenced by its specific binding mode within the SBS predicted by docking studies [32].

**Figure 2:**
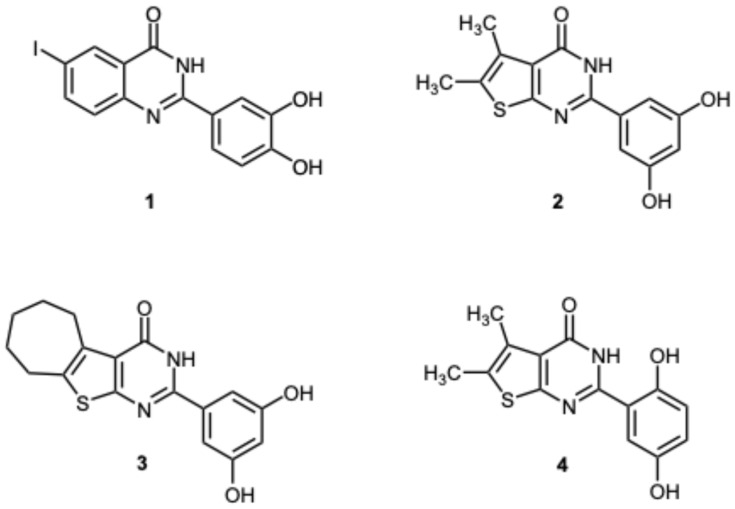
Chemical structures of and RT and IN allosteric inhibitors, dihydroxyphenyl-containing quinazolinone **1** and thienopyrimidinones **2**–**4**.

Recently, we reported the ability of various dihydroxyphenyl-containing quinazolinones [34] and thienopyrimidinones [35] to allosterically inhibit both HIV-1 RNase H and IN LEDGF/p75-dependent activities, such as compounds **1** (wt RT RNase H IC_50_ = 0.11 ± 0.01 μM), **2** (IN LEDGF/p75-dependent IC_50_ = 3.7 ± 0.73 μM), **3** (IN LEDGF/p75-dependent IC_50_ = 3.37 ± 0.48 μM) and **4** (IN LEDGF/p75-dependent IC_50_ = 85 ± 6.00 μM) (Figure 2). With respect to dihydroxyphenyl-thienopyrimidinones, we noted a significant correlation between the positioning of the hydroxyl groups on the phenyl ring and their IN inhibitory activity, with compounds **2** and **3** the most active in the IN LEDGF/p75-dependent assay [36]. We observed, for instance, that the relocation of a hydroxyl group from position 3 to position 2 can result in the inactive compound **4** (Figure 2).

Accordingly, we used compounds **2** and **4**, the most and least potent 5,6-dimethyl-thienopyrimidinones, to assess the impact of adding an extra hydroxyl group to the phenolic ring.

The present investigation involved the synthesis of several differently substituted 2-(trihydroxyphenyl)thieno[2,3-*d*]pyrimidin-4(3*H*)-ones, which were explored for their HIV-1 IN LEDGF/p75-dependent and independent inhibitory activity. Mode of action studies were also performed to define their allosteric binding as well as their ability to impact the exchange of the IN-IN subunit and affect the IN/LEDGF/p75 interaction. In silico studies were conducted to complement the in vitro findings and to identify key residue mutations involved in the interaction with the selected compounds.

## 2. Results and Discussion

As previously mentioned, the polyphenolic framework of Kuwanon-L facilitates effective interactions with IN and RT. Consequently, our investigation initially focused on 2-(trihydroxyphenyl)thienopyrimidinones, compounds previously identified as RNase H inhibitors, to determine whether their polyphenolic moieties also contribute to IN inhibition.

Every compound in this study was first assessed for its ability to affect HIV-1 IN LEDGF/p75-dependent activity. Using compounds **2** and **4** as a starting point, we investigated whether introducing a hydroxyl group at the 4-position of the benzene ring would influence the inhibitory activity. Compounds **5** and **6**, previously recognized as effective RNase H inhibitors [36], were subsequently tested. Notably, compound **6** exhibited a substantial improvement in activity (IC_50_ = 0.45 ± 0.09 μM) compared to its parent compound **4** (IC_50_ = 85 ± 6.00 μM). In contrast, a moderate decrease in inhibitory efficacy was observed upon modification of compound **2** (IC_50_ = 3.7 ± 0.73 μM) into compound **5** (IC_50_ = 8.0 ± 2.00 μM) (Table 1).

These significant findings prompted us to synthesize a range of novel 2-(trihydroxyphenyl)thienopyrimidinones, also exploring different substitutions on the thiophene ring.

**Scheme 1:**
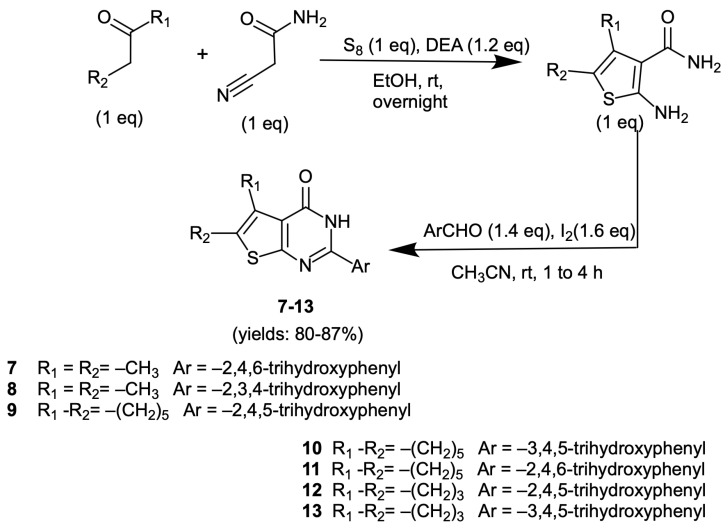
Synthesis of 2-(trihydroxyphenyl)thienopyrimidinones **7**–**13**.

The novel thienopyrimidinones **7**–**13** were synthesized in accordance with our previously established procedure for the preparation of compounds **2**–**6** [34,35,36]. The synthesis began with a Gewald reaction involving cyanoacetamide, a suitable carbonyl compound, and elemental sulfur, using diethylamine (DEA) as the base. This reaction yielded the corresponding 2-amino-4,5-disubstituted-thiophene-3-carboxamide, which subsequently underwent a direct oxidative condensation with the selected aldehyde in the presence of molecular iodine, ultimately yielding the final products (Scheme 1).

To complete the series of 5,6-dimethyl-thienopyrimidinones, we initially investigated the effects of 2,4,6- and 2,3,4-trihydroxyphenyl substitutions. Unfortunately, the resulting compounds **7** and **8** exhibited reduced potency in IN inhibition compared to compound **6**, with IC_50_ values of 6.1 ± 1.6 μM and 11.75 ± 1.85 μM, respectively.

We subsequently analyzed the introduction of different substituents on the thiophene ring. Our previous findings had already suggested that replacing the methyl groups with bulkier substituents influences the inhibitory activity of dihydrophenyl thienopyrimidinones [35].

Replacing the methyl groups with a cycloheptyl ring while retaining the 2,4,5-, 3,4,5-, and 2,4,6-trihydroxyphenyl cores, led to only a minor change in the IN inhibitory activity of resulting compounds **9**, **10** and **11** (Table 2). Specifically, in the IN LEDGF/p75 dependent assay compounds **9** and **11** exhibited either a slight reduction or comparable inhibitory activity relative to compounds **6** and **7**, respectively. Conversely, the cycloheptyl substitution proved beneficial for compound **10**, which exhibited a markedly enhanced activity relative to compound **5**. In fact, as shown in Table 1, compound **10** inhibited the IN-LEDGF/p-75 dependent activity with an IC_50_ value of 0.31 μM. These findings prompted us to investigate the potential impact of replacing the cycloheptyl ring with a cyclopentyl ring on the activity of the best derivatives **9** and **10**. Regrettably, the incorporation of a smaller ring led to compounds **12** and **13**, which exhibited a slightly reduced activity (Table 1). Nonetheless, all compounds show enhanced activity in comparison to LEDGIN-6, used as positive control.

Further investigation was conducted on the most active compounds, namely **6**, **9**, **10**, **12**, and **13**. We also opted to examine compound **5**, since the modifications on its scaffold generally improved the inhibitory activity. For a more comprehensive assessment of the effects of the tested compounds during the integration process, we analyzed their IN LEDGF/p75 independent activity (Table 2).

All tested compounds inhibited both the HIV-1 IN LEDGF/p75 dependent and independent activities within a low micromolar range, exhibiting a more potent effect than the positive control, LEDGIN-6 (Table 1 and Table 2). Compounds **6**, **9** and **12**, inhibited the HIV-1 IN LEDGF-independent activity with IC_50_ values of 0.76, 0.99 and 0.86 μM, respectively, while compounds **5**, **10** and **13**, showed an inhibition range of 1.12–5.35 μM. To provide insights into the mechanism of action of the thienopyrimidinones, we selected a subset of compounds for further investigation. Compound **6** was selected due to its outstanding activity in the HIV-1 IN LEDGF/p75-dependent assay as well as in the HIV-1 IN LEDGF/p75-independent assay, whereas compound **9** was selected for its similar activity in both assessments.

Additionally, compounds **10** and **12** were chosen for their distinct behaviors. Specifically, compound **10** showed significantly higher potency of inhibition of the IN LEDGF-dependent activity, whereas compound **12** displayed a more pronounced effect on IN inhibition in the absence of LEDGF cofactor.

Firstly, the selected compounds were assessed for their ability to disrupt the binding of HIV-1 IN to LEDGF/p75. Compounds **10** and **12** effectively inhibited the IN- LEDGF/p75 interaction, exhibiting IC_50_ values of 6.16 μM and 24.75 μM, respectively. Conversely, compound **6** showed only a minimal inhibitory effect, with an IC_50_ of 75 μM, whereas **9** was found to be inactive on the inhibition of the HIV-1 IN-LEDGF/p75 binding. Hence, we hypothesize that compounds **6** and **9** might have a different mode of action with respect to compounds **10** and **12**, which displayed a profile comparable to the positive control LEDGIN-6, as shown in Table 3.

Secondly, to further evaluate such a hypothesis, the selected compounds were also tested for their ability to affect the functional multimerization of HIV-1 IN. Notably, such an assay allows for the easy differentiation of compounds that affect the subunit exchange between the IN monomers from those that induce higher-order, aberrant IN multimerization. The results showed that, unlike LEDGIN-6, all compounds successfully inhibited IN-IN subunit exchange, with IC_50_ values ranging from 2 to 17 μM, while lacking the ability to induce higher-order, aberrant IN multimerization, typical of LEDGIN-6 (Table 3).

**Figure 3:**
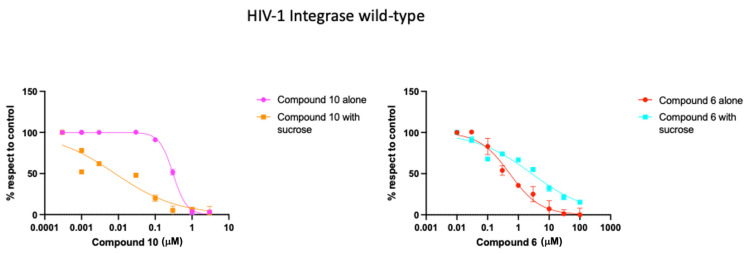
Dose-dependent curve of compounds **10** and **6** inhibitory activity on HIV-1 integrase wild-type.

The findings highlight compounds **6** and **10** as particularly significant. Compound **10** showed a marked capacity to inhibit both the HIV-1 IN-IN and IN- LEDGF/p75 interactions, whereas compound **6** not only exhibited the best IC_50_ values for both HIV-1 IN/LEDGF/p75 dependent and independent assays but also showed a reasonable inhibition of the HIV-1 IN-IN interaction. However, unlike compound **10**, it was unable to inhibit the IN-LEDGF/p75 interaction. The effect of the tested compounds on the HIV-1 IN strand-transfer activity, both with and without LEGDF/p75, as well as their influence on the interactions between HIV-1 IN-IN and HIV-1 IN-LEDGF, indicates a potential allosteric inhibition mechanism. Therefore, to further investigate the mode of action, we decided to evaluate if sucrose, which interacts with the CCD interface, could influence the inhibitory effects on the IN-LEDGF/p75-dependent activity of compounds **6** and **10**, as previously observed with LEDGINs [32]. Interestingly, the two compounds exhibited different behaviors. Compounds **10** and **6** alone show a dose-dependent inhibition (Figure 3), while sucrose alone is reported and confirmed inactive in our assay [31]. Indeed, compound **10** alone exhibited an IC_50_ value of 0.315 μM (Table 4 and Table 5), but notably, sucrose revealed a significant additive effect on its activity, increasing its inhibitory capacity by almost 16 times, yielding an IC_50_ value of 0.02 μM (Table 5 and Figure 3). Conversely, the presence of sucrose led to a reduction in the activity of compound **6** by approximately 10 times, shifting the IC_50_ value from 0.45 μM to 4.5 μM (Table 3 and Table 4 and Figure 3).

To better understand whether this distinct behavior could be related to a potential interaction with the SBS or other allosteric pockets, a series of in silico experiments were carried out.

For our in silico analysis, we used the CCD dimer structure of HIV-1 integrase in complex with sucrose (PDB ID: 3L3V), in which sucrose stabilizes the dimer interface. Potential binding pockets at this interface were mapped on both the apo and holo forms using the Schrödinger SiteMap. Pockets with SiteScore and Dscore > 1, Balance > 0.3 and volume > 225 Å^3^ were prioritized, following recommended thresholds with a slightly broader range to account for less populated allosteric conformations [37].

**Figure 4:**
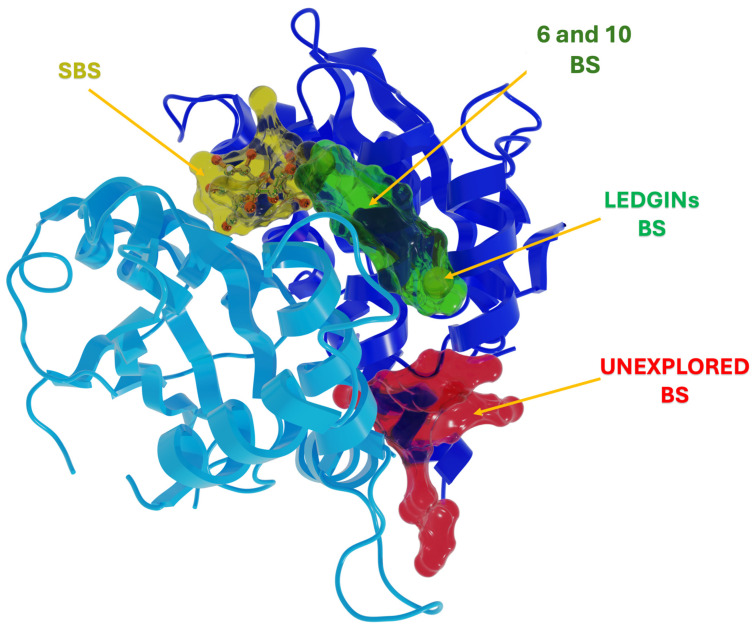
Potential binding pockets for compound **6** and **10** identified by SiteMap. SBS in yellow, LEDGINs BS in green, **6** and **10** BS in green, Unexplored BS in red.

This analysis identified two main pockets (Figure 4): (i) a region encompassing both the sucrose binding site (SBS) and the LEDGIN site, henceforth called “**6** and **10** BS”, and (ii) a deeper cavity located at the bottom of this region, hereafter referred to as the “Unexplored BS”. These two pockets were subsequently used to explore possible binding modes of compounds **6** and **10**.

Considering these results, the Glide-XP algorithm was used to carry out the docking experiments to investigate the interactions of compounds **6** and **10** with the two selected sites.

To evaluate the quality of the calculated site for the region between the SBS and the LEDGINs BS (**6** and **10** BS), two anchoring grids have been established. The first grid was based on the receptor area calculated with SiteMap for the protein in its APO form, and the second grid was designed around the crystallographic ligand of the prepared protein. For the new site to be explored, two anchoring grids were created, both based on sitemap calculations, as no reference ligands are available. One grid was built by considering the receptor area calculated with the SiteMap of the protein in its APO form, while the other was prepared using the receptor area calculated with the SiteMap of the protein in its HOLO form.

Docking simulations of compounds **6** and **10** were subsequently carried out at the two selected binding sites using both Molecular Mechanics (MM) and Quantum Mechanics (QM) approaches. The **6** and **10** BS were first investigated. The docking score was considered a fundamental parameter for assessment, facilitating a comparison of the poses of the two synthetic ligands, both in the absence and presence of sucrose, while analyzing their interactions with the receptor and their positioning relative to sucrose. Best poses docking scores indicate a favorable outcome, remaining largely unchanged both in the presence of sucrose (**6** = −6.199 Kcal/mol, **10** = −5.445 Kcal/mol) and in its absence for both ligands (**6** = −7.110 Kcal/mol, **10** = −6.730 Kcal/mol). Moreover, the conformations of **6** and **10** within the enzyme do not exhibit significant variations and remain nearly in the same position (Figure S1, Supplementary Data). This supports the effectiveness of the molecules; however, it unfortunately fails to account for the marked variation in IC_50_ values observed in the presence or absence of sucrose.

**Figure 5:**
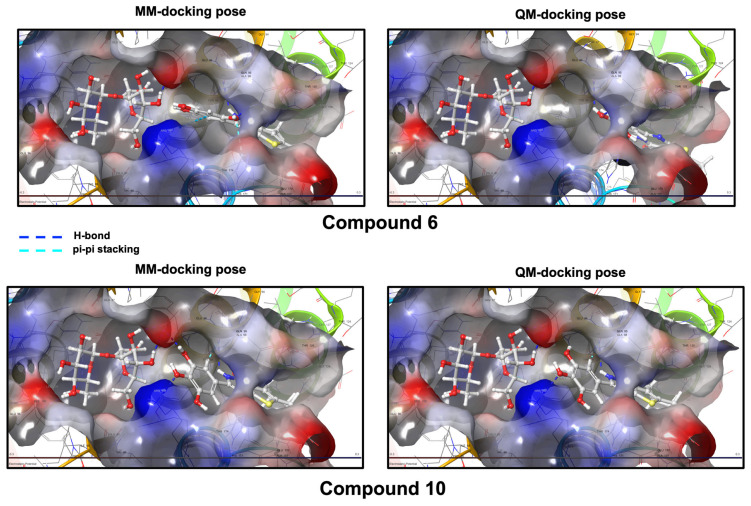
Compounds **6** and **10** docking poses according to the molecular mechanics (MM) protocol and the quantum mechanics (QM) protocol, and their ligand–receptor interactions. (**6** and **10** BS).

**Figure 6:**
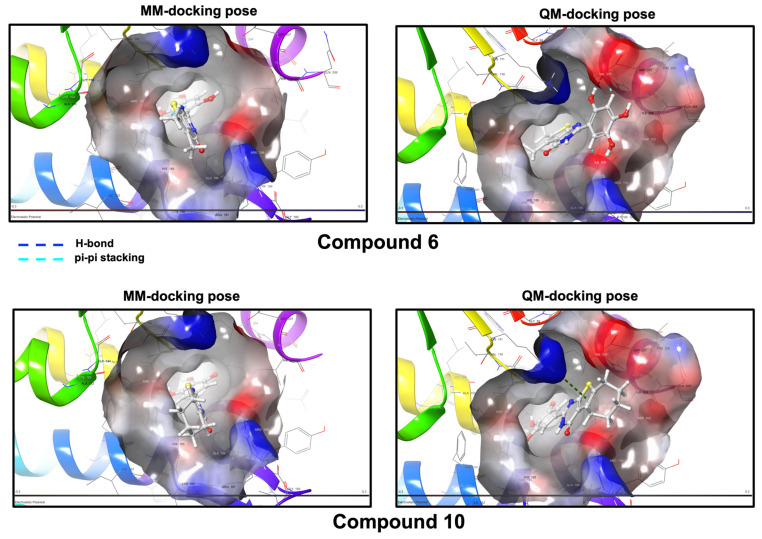
Compounds **6** and **10** docking poses according to the molecular mechanics (MM) protocol and the quantum mechanics (QM) protocol and their ligand–receptor interactions. (Unexplored BS).

To qualitatively assess how local electrostatics at the CCD interface might influence ligand binding, we refined the best docking poses of compounds **6** and **10** using a semi-empirical QM treatment of the ligands (Figure 5 and Figure 6, Figures S1 and S2 in the Supplementary Data). Within the SBS/LEDGIN region (**6** and **10** BS), compound **10** consistently exhibited slightly more favorable interaction energies compared to compound **6**, consistent with its stronger inhibition of LEDGF/p75-dependent IN activity (IC_50_ = 0.315 μM for **10** vs. 0.45 μM for **6**; Table 3 and Table 4).

In QM docking runs performed in the presence of sucrose, this trend was further supported by better docking scores for compound **10** than for compound **6** (−6.131 vs. −4.120 kcal/mol). These values are presented solely to indicate the qualitative ranking of the ligands rather than to provide precise estimates of binding free energy. The QM-refined poses also suggested that, in the presence of sucrose, compound **6** tends to sample geometries that move away from the sugar moiety, whereas compound **10** remains closely associated with sucrose. This qualitative pattern is compatible with the opposite effects of sucrose on the inhibitory activity of the two ligands on wild-type IN (improved potency for **10** and reduced potency for **6**; Table 3 and Table 4).

Docking within the deeper cavity (Unexplored BS) provided an alternative set of poses for both compounds. In MM docking, the best poses displayed satisfactory scores for both ligands (**6** = −6.730 Kcal/mol, 10 = −7.115 Kcal/mol), and subsequent QM refinement led to lower, yet still acceptable, scores (**6** = −4.027 Kcal/mol, **10** = −4.597 Kcal/mol). Overall, the interaction energies for **6** and **10** in this pocket remained in a similar range to those obtained in the SBS/LEDGIN region. On this basis, we did not attempt to rank the SBS/LEDGIN and Unexplored BS pockets solely by their docking scores. Rather, the in silico analysis is used here as a qualitative framework to propose that both regions at the CCD interface could contribute to the allosteric regulation of IN by compounds **6** and **10**. Moreover, the QM-based analysis indicates that compound **6** undergoes a positional inversion relative to the MM approach, leading to the emergence of new interactions. Likewise, compound **10** adopts a distinct orientation of its cycloheptane moiety, which promotes additional interactions between the binding pocket and the thiophene ring (Figure 6 and Figure S2 in the Supplementary Data).

Then, to confirm the involvement of the virtually predicted binding sites in the ligand-protein interaction, we performed supplementary docking experiments to pinpoint the most likely significant mutations. To identify ligand-amino acid interactions with the most favorable energy profile, a comprehensive analysis of the previously generated docking poses for each site was conducted. Specifically, we performed a per-residue energy decomposition considering all residues (amino acids and sucrose) present within 12 Å radius of the ligands. (Figures S3–S5, Supplementary Data).

Moreover, the difficulty in selecting the optimal mutant amino acid is, in certain cases, linked to the fact that the most energetically favorable interactions between the ligand and the residue do not involve the side chain, but rather the oxygen implicated in the peptide bond, a structural feature common to all amino acids. Thus, based on this observation, for the SBS, we opted to replace the Lysine 173 (Lys173) with Alanine 173 (Ala173). In fact, although Lys 173 plays a role in the interaction with sucrose through its backbone, most of its energetic contribution to the binding our compounds arise from its side chain. Replacing it with alanine eliminates these side-chain interactions while maintaining the backbone’s connections with sucrose. Therefore, Ala173 is deemed a suitable replacement, owing to its apolar side chain. Concerning the Unexplored BS, we suggest replacing Histidine 185 (His185) with Alanine 185 (Ala185). In this instance, the amino acid chosen for mutation provides a substantial energetic contribution to the protein-ligand interaction and engages with its own side chain. Alanine is confirmed as a valid substitute due to its apolar side chain.

To investigate the potential impact of the selected mutations on the binding of compounds **6** and **10** to HIV-1 IN, we executed site-directed mutagenesis, replacing Lys173 and the His185 with Ala.

**Figure 7:**
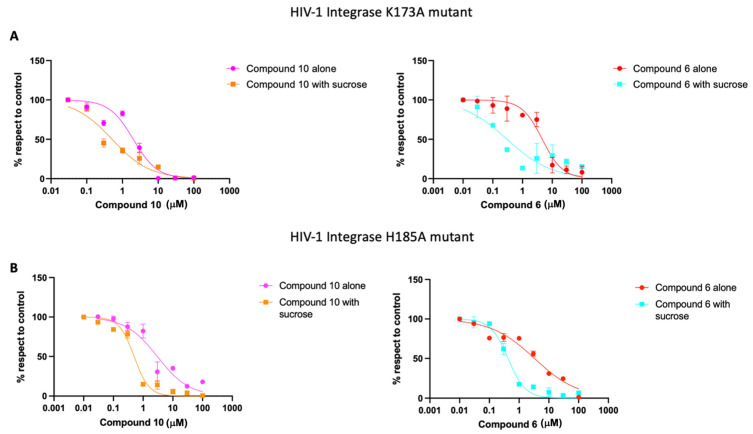
Dose-dependent curve of compounds **10** and **6** inhibitory activity on HIV-1 integrase K173A (**A**) and H185A (**B**) mutants.

Following this, we performed a biochemical assay to evaluate whether the compounds retain their inhibitory efficacy on both mutated forms of HIV-1 IN. The data presented in Table 5 and Table 6, along with Figure 7, demonstrate that although both compounds exhibit markedly reduced efficacy compared to HIV-1 IN wild-type, they still effectively inhibit the enzymatic activity of the HIV-1 IN K173A and H185A mutants in the presence of LEDGF/p75. Specifically, concerning the HIV-1 IN K173A mutation, we recorded IC_50_ values of 6.5 μM for compound **6** and 3.3 μM for compound **10** (Table 5, Figure 7A) whereas IC_50_ values of 4.64 μM for compound **6** and 2.27 μM for compound **10** were observed with the HIV-1 IN H185A mutant (Table 6, Figure 7B). Subsequently, to ascertain whether the presence of sucrose could have any effect on the inhibitory activity, as previously observed with Kuwanon-L, compounds **6** and **10** were also tested in the presence of sucrose.

Interestingly, in the presence of sucrose both compounds showed a marked increase in inhibitory activity on the K173A and H185A mutants (Table 5 and Table 6, Figure 7). For the K173A mutant, compound **6** displayed a ~33-fold increase in potency, while compound **10** showed a ~13-fold increase (Table 5 and Figure 7A). For the H185A mutant, the corresponding increases were ~9.7-fold and ~3.7-fold, respectively (Table 6 and Figure 7B). Although these effects are less straightforward than those observed for wild-type IN, they indicate that sucrose can still modulate the activity of both ligands when key residues at the CCD interface are perturbed.

Taken together with the wild-type data, these findings are qualitatively consistent with a model in which compounds **6** and **10** interact with allosteric pockets located in the SBS/LEDGIN region and in the neighboring Unexplored BS, and in which sucrose binding at the CCD interface can differentially influence their inhibitory profiles. However, we note that these functional data do not uniquely define a single binding mode, and the proposed model should be regarded as a working hypothesis that integrates biochemical and docking results.

We also evaluated the effect of compounds **6** and **10** on viral replication. Regrettably, both compounds were not able to inhibit viral replication and were devoid of cytotoxicity.

## 3. Materials and Methods

### 3.1. Chemistry

#### 3.1.1. General

The reagents and solvents utilized in this study were sourced from commercial suppliers and utilized without additional purification. The progress of the reaction was tracked using thin-layer chromatography, using Merck silica gel 60 pre-coated plates with a fluorescent indicator F254 (0.25 mm) (Merck, Sigma Aldrich, Milan, Italy). The ^1^H and ^13^C NMR spectra were acquired on a Bruker Avance III™ HD 600 MHz spectrometer (Bruker, Billerica, MA, USA), operating at frequencies of 600 and 151 MHz, respectively. Negative ESI-MS spectra were recorded on a high-resolution LTQ Orbitrap Elite™ mass spectrometer (Thermo Fisher Scientific, Waltham, MA, USA). The solutions were infused into the ESI source at a flow rate of 5.00 μL/min. Spectra were recorded with a resolution of 240.000 (FWHM). Instrument conditions were as follows: spray voltage 4000 and 5000 V for positive and negative ionization mode, respectively, capillary temperature 275 °C, sheath gas 12 (arbitrary units), auxiliary gas 3 (arbitrary units), sweep gas 0 (arbitrary units), probe heater temperature 50 °C.

#### 3.1.2. General Synthetic Procedure for Synthesis of 2-(Trihydroxyphenyl)Thieno[2,3-d]Pyrimidin-4(3H)-Ones 7–13

Initially, we started with the synthesis of the 2-amino-4,5-disubstituted-thiophene-3-carboxamide intermediates that was conducted as follows. A mixture containing cyanoacetamide (25 mmol), the selected ketone (25 mmol), diethylamine (27.5 mmol), sulfur (25 mmol), and dry ethanol (20 mL) was stirred overnight at room temperature. Once the reaction was complete, it was quenched with 150 mL of water and extracted five times with a 3:1 mixture of ethyl acetate and ethanol. The organic layer was dried over sodium sulfate, filtered, and the solvent was evaporated under vacuum, resulting in a sticky oil or a solid. The crude product was further purified by crystallization from ethanol, resulting in the desired 2-amino-4,5-disubstituted-thiophene-3-carboxamide. Subsequently, the 2-amino-4,5-disubstituted-thiophene-3-carboxamide (2 mmol), molecular iodine (2.61 mmol), the suitable aromatic aldehyde (2.44 mmol), were stirred in acetonitrile (50 mL) at room temperature until completion indicated by TLC (1 to 4 h). Following the reaction, the mixture was treated with 60 mL of a 5% sodium thiosulfate solution to quench the reaction, and the resulting solid was isolated and collected through vacuum filtration. The crude product was subsequently washed with a petroleum ether/ethyl acetate mixture (1:1) and recrystallized from ethanol, yielding the final products **7**–**13**.

#### 3.1.3. Characterization Data of All the Tested Compounds

5,6-Dimethyl-2-(3,4,5-trihydroxyphenyl)thieno[2,3-d]pyrimidin-4(3*H*)-one (**5**). Pale yellow solid. M.p. 329–330 °C. Yield = 529 mg (87%). NMR spectra are consistent with those previously reported [34]. IR (mineral oil), cm^−1^: 3750, 3302, 1648,1560, 1430. ^1^H NMR (600 MHz, DMSO) δ 12.09 (s, 1H), 9.15 (s, 2H), 8.86 (s, 1H), 7.13 (s, 2H), 2.40 (s, 3H), 2.35 (s, 3H) ppm. ^13^C NMR (151 MHz, DMSO) δ 163.16, 159.54, 152.88, 146.35, 137.38, 129.10, 128.77, 122.36, 121.29, 107.54, 13.33, 13.08 ppm. HRMS calculated for C_14_H_11_N_2_O_4_S 303.0518, found [M-H]^−^ 303.0449.

5,6-Dimethyl-2-(2,4,5-trihydroxyphenyl)thieno[2,3-d]pyrimidin-4(3H)-one (**6**). Light brown solid. M.p. > 360 °C. Yield = 505 mg (83%). NMR spectra are consistent with those previously reported [36]. IR (mineral oil), cm^−1^: 3680, 3310, 1649, 1534, 1406. ^1^H NMR (600 MHz, DMSO) δ 11.75 (s, 1H), 9.87 (s, 1H), 8.67 (s, 1H), 7.61 (s, 1H), 6.44 (s, 1H), 2.39 (s, 3H), 2.35 (s, 3H). ^13^C NMR (151 MHz, DMSO) δ 166.50, 156.89, 152.74, 150.66, 134.24, 132.46, 121.32, 120.33, 118.67, 117.73, 115.79, 114.44, 13.27, 13.09 ppm. HRMS calculated for C_14_H_11_N_2_O_4_S 303.0518, found [M-H]^−^ 303.0427.

5,6-Dimethyl-2-(2,4,6-trihydroxyphenyl)thieno[2,3-d]pyrimidin-4(3*H*)-one (**7**). Pale yellow solid. M.p. 335–337 °C. Yield = 517 mg (85%). IR (mineral oil), cm^−1^: 3700, 3323, 1710, 1612, 1462. ^1^H NMR (600 MHz, DMSO) δ 11.62 (s, 1H), 10.14 (s, 1H), 5.82 (s, 2H), 2.29 (s, 3H), 2.05 (s, 3H) ppm. ^13^C NMR (151 MHz, DMSO) δ 166.78, 164.56, 164.24, 161.97, 153.69, 146.74, 145.19, 132.71, 131.77, 101.74, 13.63, 12.88 ppm. HRMS calculated for C_14_H_11_N_2_O_4_S 303.0518, found [M-H]^−^ 303.0452.

5,6-Dimethyl-2-(2,3,4-trihydroxyphenyl)thieno[2,3-d]pyrimidin-4(3*H*)-one (**8**). Beige solid. M.p. > 360 °C. Yield = 523 mg (86%). IR (mineral oil), cm^−1^: 3690, 3335, 1657, 1547, 1459. ^1^H NMR (600 MHz, DMSO) δ 12.20 (s, 1H), 9.79 (s, 1H), 8.60 (s, 1H), 7.77 (s, 1H), 7.58 (d, *J* = 8.6 Hz, 1H), 6.97 (d, *J* = 8.6 Hz, 1H), 2.40 (s, 3H), 2.37 (s, 3H) ppm. ^13^C NMR (151 MHz, DMSO) δ 167.68, 164.56, 164.24, 161.97, 153.69, 146.74, 132.71, 131.77, 129.20, 126.97, 101.74, 13.63, 12.88 ppm. HRMS calculated for C_14_H_11_N_2_O_4_S 303.0518, found [M-H]^−^ 303.0444.

2-(2,4,5-Trihydroxyphenyl)-3,5,6,7,8,9-hexahydro-4*H*-cyclohepta[4,5]thieno[2,3-*d*]pyrimidin-4-one (**9**). Light brown solid. M.p. 339–340 °C. Yield = 564 mg (82%). IR (mineral oil), cm^−1^: 3690, 3346, 1709, 1658, 1547, 1460. ^1^H NMR (600 MHz, DMSO) δ 11.74 (s, 1H), 9.89 (s, 1H), 8.69 (s, 1H), 7.60 (s, 1H), 6.44 (s, 1H), 3.26–3.24 (m, 2H), 2.85–2.79 (m, 2H), 1.87–1.83 (m, 2H), 1.66–1.58 (m, 4H) ppm. ^13^C NMR (151 MHz, DMSO) δ 166.64, 162.96, 162.61, 160.45, 147.02, 133.81, 132.93, 132.15, 128.88, 127.63, 108.68, 103.69, 13.67, 13.30, 13.08, 13.02 ppm. HRMS calculated for C_17_H_15_N_2_O_4_S 343.0831, [M-H]^−^ found 343.0763.

2-(3,4,5-Trihydroxyphenyl)-3,5,6,7,8,9-hexahydro-4*H*-cyclohepta[4,5]thieno[2,3-*d*]pyrimidin-4-one (**10**). Light green solid. M.p. > 360 °C. Yield = 585 mg (85%). IR (mineral oil), cm^−1^: 3700, 3342, 1649, 1547, 1460. ^1^H NMR (600 MHz, DMSO) δ 12.09 (s, 1H), 9.14 (s, 2H), 8.85 (s, 1H), 7.12 (s, 2H), 3.27–3.26 (m, 2H), 2.83–2.81 (m, 2H), 1.87–1.84 (m, 2H), 1.66–1.57 (m, 4H) ppm. ^13^C NMR (151 MHz, DMSO) δ 162.39, 159.78, 152.73, 146.35, 137.34, 136.85, 135.93, 122.36, 121.08, 107.52, 32.48, 29.56, 27.86, 27.72, 27.39 ppm. HRMS calculated for C_17_H_15_N_2_O_4_S 343.0831, found [M-H]^−^ 343.0735.

2-(2,4,6-Trihydroxyphenyl)-3,5,6,7,8,9-hexahydro-4*H*-cyclohepta[4,5]thieno[2,3-*d*]pyrimidin-4-one (**11**). Light brown solid. M.p. 340–342 °C. Yield = 571 mg (83%). IR (mineral oil), cm^−1^: 3695, 3349, 1711, 1640, 1547, 1461. ^1^H NMR (600 MHz, DMSO) δ 5.81 (s, 2H), 2.77–2.72 (m, 2H), 2.64–2.59 (m, 2H), 1.81–1.79 (m, 2H), 1.62–1.54 (m, 4H), ppm. ^13^C NMR (151 MHz, DMSO) δ 166.64, 162.96, 162.61, 162.11, 157.42, 133.81, 132.93, 132.15, 130.42, 127.63, 112.70, 108.69, 13.67, 13.30, 13.08, 13.02 ppm. HRMS calculated for C_17_H_15_N_2_O_4_S 343.0831, found [M-H]^−^ 343.0829.

2-(2,4,5-Ttrihydroxyphenyl)-3,5,6,7-tetrahydro-4*H*-cyclopenta[4,5]thieno[2,3-*d*]pyrimidin-4-one (**12**). Light orange solid. M.p. 344–346 °C. Yield = 506 mg (80%). IR (mineral oil), cm^−1^: 3700, 3380, 1659, 1542, 1461. ^1^H NMR (600 MHz, DMSO) δ 11.61 (s, 1H), 10.11 (s, 1H), 7.48 (s, 1H), 5.97 (s, 1H), 2.63–2.61 (m, 2H), 1.63–1.58 (m, 4H) ppm. ^13^C NMR (151 MHz, DMSO) δ 164.32, 164.05, 161.88, 145.37, 139.10, 133.82, 133.79, 105.14, 101.75, 94.77, 94.59, 32.22, 28.42, 28.14 ppm. HRMS calculated for C_15_H_11_N_2_O_4_S 315.0518, found [M-H]^−^ 315.0443.

2-(3,4,5-Trihydroxyphenyl)-3,5,6,7-tetrahydro-4*H*-cyclopenta[4,5]thieno[2,3-*d*]pyrimidin-4-one (**13**). Light brown solid. M.p. > 360 °C. Yield = 544 mg (86%). IR (mineral oil), cm^−1^: 3747, 3306, 1658, 1534, 1462. ^1^H NMR (600 MHz, DMSO) δ 12.19 (s, 1H), 9.16 (s, 1H),7.14 (s, 2H), 2.93–2.90 (m, 4H), 2.42–2.37 (m, 2H) ppm. ^13^C NMR (151 MHz, DMSO) δ 169.31, 158.99, 152.86, 146.36, 139.99, 137.42, 137.01, 122.40, 118.11, 107.57, 29.48, 29.16, 27.86 ppm. HRMS calculated for C_15_H_11_N_2_O_4_S 315.0518, found [M-H]^−^ 315.0446.

### 3.2. In Silico Studies

#### 3.2.1. Protein Preparation

The crystallographic structure of the CCD domain in complex with sucrose (PDB ID: 3L3V) was retrieved from the RCSB Protein Data Bank. This structure is biologically relevant in its dimeric form and co-crystallized with sucrose, which helps lower the overall energy of the dimer, thereby stabilizing the complex. All residual crystallization components (including cadmium ions and sulfates) were removed, and cadmium was replaced by magnesium ions (Mg^2+^) to reflect the biologically active metal cofactor for this protein.

The tools of Schrödinger suite were used to carry out the in silico experiments (Schrödinger release 2022-4, Schrödinger, LLC, New York, NY, USA).

Subsequently, the protein was preprocessed using the Protein Preparation Wizard, which included:

- assignment of bond orders, particularly creating zero-order bonds to metals;
- addition of missing hydrogen atoms lost during crystallization;
- automatic creation of disulfide bridges;
- conversion of selenomethionine to methionine;
- rebuilding of missing loops and chain termini using the Prime module.
- Free N- and C-terminal residues were capped with acetyl (ACE) and methylamide (NMA) groups, respectively.

Water molecules within 5 Å of the crystallized ligand were removed, and the possible protonation states of nonstandard residues were generated in Epik (pH 7.0 ± 2.0).

All hydrogen-bonding networks were then optimized using the PropKa tool (pH 7.0), ensuring correct protonation states for titratable side chains, and water orientations in the binding site were refined. A restrained minimization was performed using the OPLS3e force field, converging heavy atoms to a maximum displacement threshold of 0.3 Å.

#### 3.2.2. Binding Site Exploration

The prepared apo- and holo-forms of the protein (the latter retaining the co-crystallized sucrose) were analyzed with SiteMap to identify potential binding pockets. Default parameters were employed, with the requirement that each site contain at least six site points, and the best 10 sites were reported. A more restrictive hydrophobic definition was chosen to better characterize potential binding pockets. Key SiteMap parameters (SiteScore ≥ 1, Dscore ≥ 1, Balance > 0.3, volume > 225 Å^3^) were used to evaluate and compare the identified sites.

- Crystallographic Ligand

Sucrose (PubChem ID: 5988) was downloaded from the PubChem database and prepared using LigPrep with the OPLS3e force field. Possible protonation states at pH 7.0 ± 2.0 and tautomeric forms were generated with Epik, preserving the experimentally determined chirality of the sugar.

- Synthetic Ligands

Two synthetic ligands of interest, labeled **6** and **10**, were sketched with the 2D editor in the Schrödinger suite and similarly prepared with LigPrep. As with sucrose, all possible ionization and tautomeric states were generated at pH 7.0 ± 2.0 using Epik, and stereoisomeric configurations were considered where applicable. The OPLS3e force field was used for all ligand preparation steps.

#### 3.2.3. Receptor Grid Generation

For each binding site (**6** and **10** BS, and unexplored BS), two grids were generated to account for differences between the apo and holo forms. In the **6** and **10** BS, one grid was centered on the SiteMap -derived pocket from the apo structure, and the second grid was centered on the co-crystallized sucrose position in the holo structure. For the unexplored BS, two grids were similarly derived from the SiteMap predictions in the apo and holo structures, respectively. In each case, a cubic box of suitable dimensions was placed around the binding site. The van der Waals scaling factor was set to 1.0, and a partial charge cutoff of 0.25 was applied.

#### 3.2.4. Docking Protocol

Docking simulations were performed with Glide in Extra Precision (XP) mode. Flexible ligand sampling was enabled, including nitrogen inversion, ring conformations, and torsion bias sampling for predefined functional groups. The following parameters were applied in all docking runs:

- Van der Waals radius scaling factor (for ligand): 0.80
- Partial charge cutoff: 0.15
- Penalization of states generated by Epik
- Post-docking energy minimization
- Recording interaction scores for all residues within a 12 Å cutoff from the ligand

For the **6** and **10** BS, docking simulations were performed both in the presence of the crystallized sucrose (to investigate potential stacking or secondary site occupancy) and in its absence (to study a vacant pocket). In the Unexplored BS, the two grids (apo- and holo-derived) were used without any reference ligand.

#### 3.2.5. Quantum Mechanical Docking Approach

To gain further insight into binding behaviors, an additional docking protocol incorporating quantum mechanical (QM) calculations was carried out. Following initial XP Glide docking, semiempirical methods (NDDO module) were employed to recalculate partial charges on the ligands via the Coulson–Fischer approach. The ligands were then re-docked with the updated charge distributions using XP mode in Glide. Final poses were selected based on combined Coulomb–van der Waals considerations.

#### 3.2.6. Mutational Studies

To discriminate the involvement of each binding site in ligand inhibition more definitively, we performed in silico point mutations of key residues identified as having a significant energetic contribution in protein–ligand interactions. Residues were chosen by examining the energetic contributions around 12 Å of the bound ligand, focusing on side chains forming strong hydrogen bonds or favorable electrostatic/hydrophobic contacts.

(1) The **6** and **10** BS
  (a) K173A: Lys173 demonstrated a strong energetic contribution to binding, though it also interacts with sucrose. Alanine was chosen to remove the charged side chain while minimizing steric perturbations.
  (b) Q95I: Gln95 contributed favorably to binding in 10–protein interactions and did not form optimal contacts with sucrose. Replacement with isoleucine was tested to observe significant energetic and pose changes.
  (c) Y99A: Tyr99 showed a relatively lower overall energy contribution but primarily interacted through its aromatic side chain. Mutation to alanine was chosen to reduce potential π-interactions and hydrogen bonding.
(2) The unexplored BS
  (a) H185A: His185 exhibited a favorable interaction predominantly through its side chain. Ala185 was selected to remove the polar imidazole group and assess binding changes.

Each mutated protein structure was subjected to an XP Glide docking protocol identical to that described above. For selected complexes, binding free energies were evaluated through Prime MM-GBSA, with a VSGB solvation model, the OPLS3e force field, and full-protein flexibility during minimization. Comparison of docking scores and free energies of the wild type versus mutant complexes helped clarify which binding site is most relevant for the synthetic ligands of interest.

### 3.3. Biology

#### 3.3.1. Site-Direct Mutagenesis on HIV-1 INs

The commercial QuikChange mutagenesis Kit (Agilent Technologies Inc., Santa Clara, CA, USA) were used to introduce the selected substitutions on HIV-1 IN wt plasmid and the correct presence of alanine in 173 and 185 position were evaluated in the electropherogram provided by Bio-Fab, after the sequencing.

#### 3.3.2. Proteins Production

HIV-1 IN wt, K173A and H185A mutant proteins were expressed in *Escherichia coli* strain BL21 (DE3) after transformation with a plasmid containing protein gene. Recombinant proteins were then purified using a Ni-Sepharose column and a heparin column and stored as previously described [1]. LEDGF/p75 with 6-his and FLAG tags were purified with a heparin column and a Superdex 200 GL (Merck Sigma Aldrich, Milan, Italy) column previously described [30].

#### 3.3.3. HTRF LEDGF/p75-Dependent and -Independent Assays

The IN LEDGF/p75-dependent and indepentent assay was performed for the evaluation of 3′-processing and strand-transfer IN reactions inhibition in the presence/absence of recombinant LEDGF/p75 protein, as previously described [38].

The same assay in the presence of HIV-1 IN wt, K173A and H185A mutant proteins, was used to evaluate the effect of sucrose in the presence of selected compounds.

#### 3.3.4. HTRF-Based IN-LEDGF/p75 Binding Assay

The IN-LEDGF/p75 binding was evaluated using a FRET method, in which His-IN was pre-incubated with test compounds in a mix buffer previously described, for 30 min at room temperature [39]. After the addition of FLAG-LEDGF/p75 and a mixture of anti-His6-XL665 and anti-FLAG-EuCryptate antibodies, the reaction was read after 4 h at 4 °C, using 314 nm for excitation wavelength and 668 and 620 nm for the wavelength of the acceptor and donor emission, respectively, with a Perkin Elmer Victor 3 plate reader (Perkin Elmer, Waltham, MA, USA), and the HTRF signal calculated by the emission ratio 665 nm/620 nm multiplied by 10.000.

#### 3.3.5. The HTRF-Based Assay to Monitor Higher-Order, Aberrant IN Multimerization or IN-IN Subunit Exchange

HIV-1 His and FLAG-tagged INs were mixed in a mix buffer previously described [33] and compounds added to the mixture and incubated for 2.5 h at room temperature. At the end of the time, a mixture of anti-His6-XL665 and anti-FLAG-EuCryptate antibodies were added to the reaction and, after an incubation time of 3 h, the HTRF signal was recorded as described in the previous paragraph.

#### 3.3.6. Antiviral Activity

Antiviral activity of selected thienopyrimidinones was determined via the 2,3-bis[2-methoxy-4-nitro-5-sulfophenyl]-5-[(phenylamino)-carbonyl]-2*H* tetrazolium hydroxide (XTT)-based cell viability assay [40], using the HIV-1RF isolate and human T-cell line CEM-SS.

## 4. Conclusions

In summary, all the tested compounds were found to be significantly more effective than LEDGIN-6, the control compound, in both the HIV-1 IN LEDGF/p75-dependent and independent activity assays. These findings suggest a potential allosteric mechanism of action. Accordingly, compounds **6**, **9**, **10**, and **12** underwent further investigation, revealing their ability to interfere with IN-IN subunit exchange and modulate the interaction between IN and LEDGF/p75. However, none succeeded in inducing multimerization, unlike LEDGINs, indicating an alternative and innovative mode of action.

Subsequent in silico studies performed on the two most representative compounds **6** and **10**, suggest that a pocket located between the SBS and the LEDGIN site, together with a deeper cavity at its bottom (Unexplored BS), may represent relevant allosteric regions for ligand binding. The mutational analysis at Lys173 and His185, in the presence and absence of sucrose, provides functional support for this working model, although it does not allow us to unambiguously assign a single binding mode.

Overall, the combination of sucrose-dependent inhibition, the K173A and H185A mutants, and the IN–IN/IN–LEDGF/p75 interaction assays is consistent with an allosteric mechanism in which compounds **6** and **10**, and potentially other agents, modulate integrase activity by engaging partially overlapping sites at the CCD interface. The docking and QM calculations offer a qualitative structural framework for this hypothesis, while future structural and biophysical studies will be required to define the exact binding mode of these ligands.

## Figures and Tables

**Table 1:** Effect of **5**, **6**, **9**, **10**, **11**, **12** and **13** on the HIV-1 IN LEDGF/p75-dependent activity with respect to LEDGIN-6 as reference compound.

Compound	^a^ IC_50_ (μM)
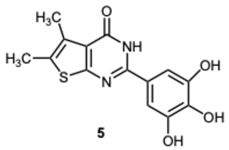	8.0 ± 2.00
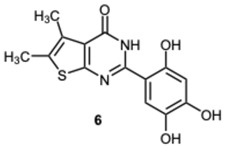	0.45 ± 0.09
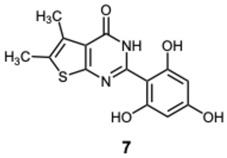	6.1 ± 1.6
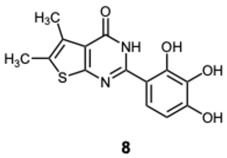	11.75 ± 1.85
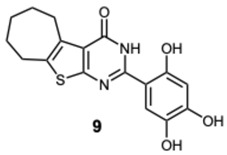	0.97 ± 0.035
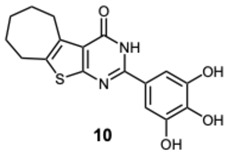	0.31 ± 0.035
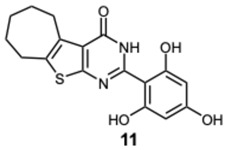	5.2 ± 1.20
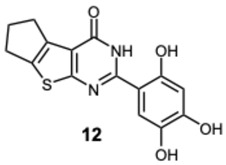	1.96 ± 0.125
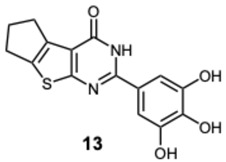	0.87 ± 0.045
LEDGIN-6	9.0 ± 2.0

^a^ Compound concentration required to inhibit the HIV-1 IN catalytic activities by 50% in the presence of LEDGF/p75. Values are expressed as means ± the SE from three experiments.

**Table 2:** Effect of compounds **5**, **6**, **9**, **10**, **12** and **13** on the HIV-1 IN activity in the absence of LEDGF/p75, with respect to LEDGIN-6 as reference compound.

Compound	^a^ IC_50_ (μM)
**5**	5.35 ± 0.55
**6**	0.76 ± 0.01
**9**	0.99 ± 0.09
**10**	3.6 ± 0.39
**12**	0.86 ± 0.14
**13**	1.12 ± 0.22
LEDGIN-6	10.0 ± 2.0

^a^ Compound concentration required to inhibit the HIV-1 IN catalytic activities by 50% in the absence of LEDGF/p75. Values are expressed as means ± the SE from three experiments.

**Table 3:** Effect of compounds **6**, **9**, **10**, and **12** on the HIV-1 IN activities with respect to LEDGIN-6 as reference compound.

Compound	IC_50_ IN LEDGF/p75-Dependent Integration ^a^ (μM)	IC_50_ IN LEDGF/p75-Independent Integration ^b^ (μM)	IC_50_ IN/LEDGF/p75 Binding ^c^ (μM)	MI_50_ IN Multimerization ^d^ (μM)	IC_50_ IN-IN Subunit Exchange ^e^ (μM)
**6**	0.45 ± 0.09	0.76 ± 0.01	75 ± 12	-	5.45 ± 0.05
**9**	0.965 ± 0.035	0.99 ± 0.09	>100 (100%)	-	6.35 ± 1.35
**10**	0.315 ± 0.035	3.6 ± 0.39	6.16 ± 0.98	-	2.05 ± 0.35
**12**	1.96 ± 0.125	0.86 ± 0.14	24.75 ± 1.75	-	17.3 ± 1.70
LEDGIN-6	9.0 ± 2.0	10.0 ± 0.5	10.0 ± 0.4	10.0 ± 1.0	>100 (100%)

^a^ Compound concentration required to inhibit the HIV-1 IN catalytic activities by 50% in the presence of LEDGF/p75. ^b^ Compound concentration required to inhibit the HIV-1 IN catalytic activities by 50% in absence of LEDGF/p75. ^c^ Compound concentration required to inhibit the HIV-1 IN/LEDGF/p75 binding by 50%. ^d^ Compound concentration required to inhibit the multimerization increase by 50%. ^e^ Compound concentration required to inhibit the HIV-1 IN-IN subunit exchange by 50%. Values are expressed as means ± the SE from three experiments.

**Table 4:** Effect of compounds **10** and **6** on HIV-1 IN (LEDGF/p75 dependent activity) in the absence and presence of sucrose.

Compound	HIV-1 IN ^a^ IC_50_ (μM)	
	Alone	With Sucrose
**10**	0.315 ± 0.035	0.02 ± 0.001
**6**	0.45 ± 0.09	4.50 ± 0.002

^a^ Compound concentration required to inhibit the HIV-1 IN catalytic activities by 50%. Values are expressed as means ± the SE from three experiments.

**Table 5:** Effect of compounds **6** and **10** on HIV-1 IN K173A mutant (LEDGF/p75 dependent activity) in the presence and absence of sucrose.

Compound	HIV-1 IN K173A Mutant ^a^ IC50 (μM) Alone	HIV-1 IN K173A Mutant ^a^ IC_50_ (μM) with Sucrose	Fold of Increase of Potency
**6**	6.50 ± 1.70	0.20 ± 0.00013	32.5
**10**	3.30 ± 0.86	0.25 ± 0.003	13.2

^a^ Compound concentration required to inhibit the HIV-1 IN K173A mutant catalytic activities by 50%. Values are expressed as medians ± the SE from three experiments.

**Table 6:** Effect of compounds **6** and **10** on HIV-1 IN H185A mutant (LEDGF/p75 dependent activity) in the presence and absence of sucrose.

Compound	HIV-1 IN H185A Mutant ^a^ IC_50_ (μM) Alone	HIV-1 IN H185A Mutant ^a^ IC_50_ (μM) with Sucrose	Fold of Increase of Potency
**6**	4.64 ± 0.70	0.48 ± 0.08	9.7
**10**	2.27 ± 0.43	0.61 ± 0.022	3.7

^a^ Compound concentration required to inhibit the HIV-1 IN H185A mutant catalytic activities by 50%. Values are expressed as medians ± the SE from three experiments.

## Data Availability

Data will be made available on request.

## Supplementary Materials

The following supporting information can be downloaded at https://www.mdpi.com/article/10.3390/molecules30244709/s1, (1) ^1^H and ^13^C NMR spectra of compounds **5**–**13**. (2) HRMS spectra of compounds **6** and **10**. (3) Figure S1. Interactions of compounds **6** and **10** with the region between the SBS and the LEDGINs BS (**6** and **10** BS) in the presence of sucrose were identified by MM and QM approaches. (4) Figure S2. Interactions of compounds **6** and **10** and the Unexplored BS, identified by MM and QM approaches. (5) Figure S3. Estimation of the contributions and energy gains of all amino acids located within a 12 Å radius of the ligands. [pocket in between the SBS and the LEDGINs BS (**6** and **10** BS) without sucrose]. (6) Figure S4. Estimation of the contributions and energy gains of all amino acids and sucrose located within a 12 Å radius of the ligands. [pocket in between the SBS and the LEDGINs BS (**6** and **10** BS) without sucrose]. (7) Figure S5. Estimation of the contributions and energy gains of all amino acids located within a 12 Å radius of the ligands. (Unexplored BS).

## Abbreviations

The following abbreviations are used in this manuscript:

IN	Integrase
DNA	Deoxyribonucleic acid
ssRNA	Single strand RNA
NTD	N-terminal domain
CCD	Central catalytic core domain
CTD	C-terminal domain
LEDGF/p75	Lens-epithelium-derived growth factor
INSTIs	Strand transfer inhibitors
ALLINIs	Allosteric integrase inhibitors
IBD	LEDGF/p75 Integrase Binding Pocket
SBS	Sucrose binding site
RT	ReverseTranscriptase
RNase H	Ribonuclease H
RdRp	RNA-dependent DNA polymerase
DEA	Diethylamine
MM	Molecular Mechanical
QM	Quantum Mechanical
ACE	Acetyl
DMSO	N,N-Dimethylsulfoxide
XP	Extra Precision
LEDGINs	LEDGF/p75 inhibitors
TLC	Thin-Layer Chromatography
NMA	Methylamide
ALLINIs	Allosteric HIV-1 integrase inhibitors
LYS	Lysine
ALA	Alanine
HIS	Histidine
NDDO	Neglect of diatomic differential overlap
GLN	Glutamine
TYR	Tyrosine
MM/GBSA	Generalised born and surface area solvation
