# Supplementary material for: 2-(Trihydroxyphenyl)thienopyrimidinones as Key Scaffolds for Targeting a Novel Allosteric Site of HIV-1 Integrase
Source: Molecules. 2025 Dec 9;30(24):4709. doi: 10.3390/molecules30244709 (PMC12735844; doi:10.3390/molecules30244709)
Supplement: Supplementary file 1 [file molecules-30-04709-s001.zip › molecules-3976207-supplementary.pdf]

2-(Trihydroxyphenyl)thienopyrimidinones as Key Scaffolds for Targeting a Novel Allosteric Site of HIV-1 Integrase.

Graziella Tocco[1,]*, Antonio Laus[1,2], Mattia Casula[1], Pierluigi Caboni[1], John A. Beutler[3], Enzo Tramontano[1], Francesca Esposito[1,]*

[1] Department of Life and Environmental Sciences, Cittadella Universitaria di Monserrato, 09042, Monserrato (Italy).
[2] Center for Advanced Studies Research and Development in Sardinia (CRS4), 09050 Pula, Cagliari, Italy (present address).
[3] Molecular Targets Program, National Cancer Institute, Frederick, MD, United States
Correspondence: toccog@unica.it; francescaesposito@unica.it

TABLE OF CONTENTS

-^{1}H and ^{13}C NMR spectraof compounds **5-13**………………………………………………………………….. pp.2-19
-HRMS spectra of compounds **6** and **10**…………………………… ……………………………………………pp.20-21
- Interactions of compounds **6** and **10** with the region between the SBS and the LEDGINs BS (**6** and **10** BS) in the presence of sucrose identified by MM and QM approaches (Figure S1)……………………………… ……………….…….p. 22
-Interactions of compounds **6** and **10** and the Unexplored BS, identified by MM and QM approaches (Figure S2)….p.23
- Estimation of the contributions and energy gains of all amino acids located within a 12Å radius of the ligands. [pocket in between the SBS and the LEDGINs BS (**6** and **10** BS) without sucrose)] (Figure S3)……………………..p. 24
- Estimation of the contributions and energy gains of all amino acids and sucrose located within a 12Å radius of the ligands. [pocket in between the SBS and the LEDGINs BS (**6** and **10** BS)] (Figure S4)……………………...... p. 25
- Estimation of the contributions and energy gains of all amino acids located within a 12Å radius of the ligands. (Unexplored Binding Site) (Figure S5)…………………………………………………………………......... p. 26

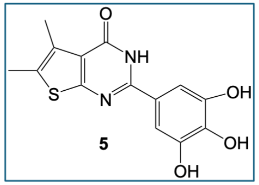

O
NH
S
N
OH
OH
OH

5

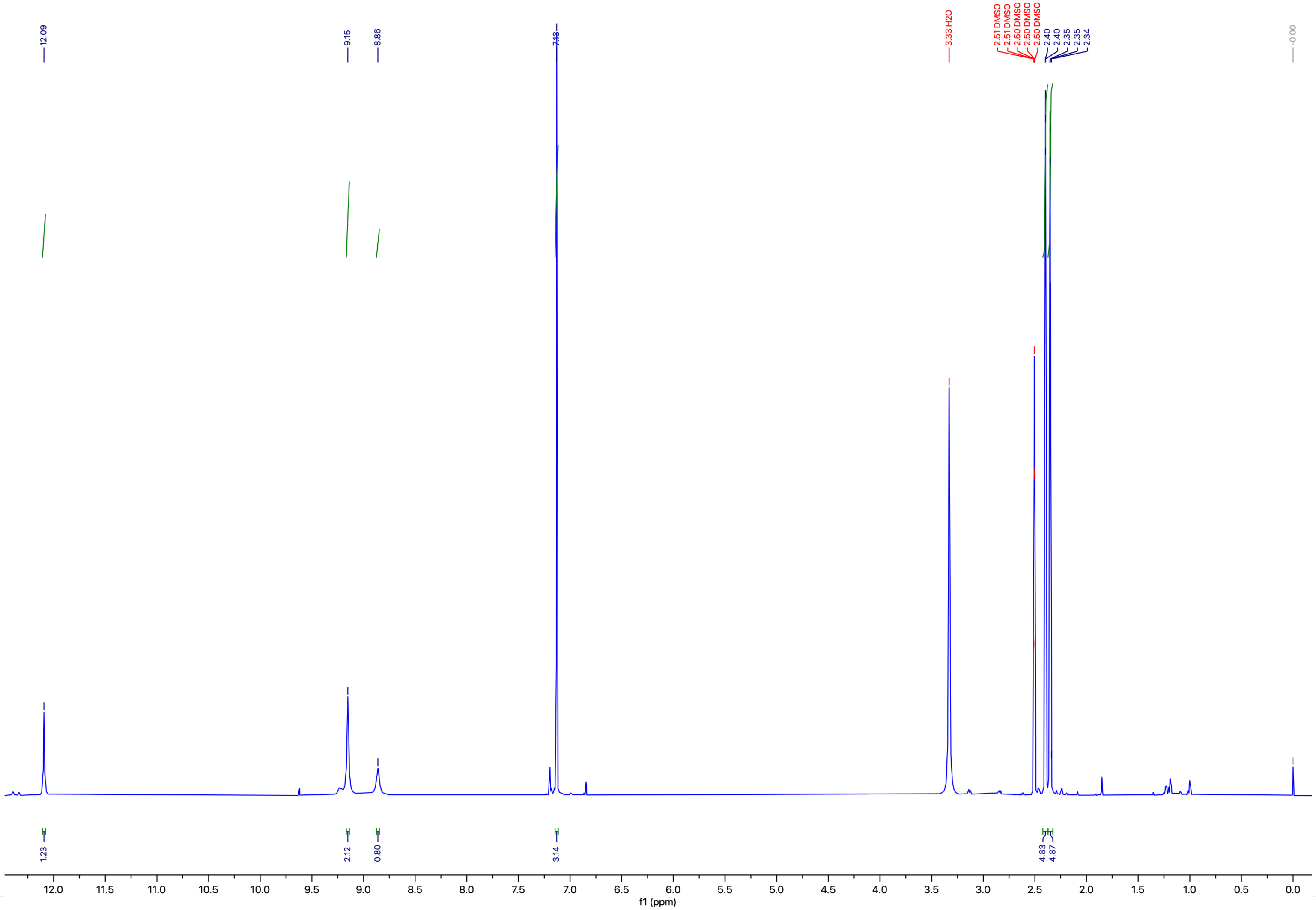

12.09
9.15
8.86
7.13
3.33 H2O
2.51 DMSO
2.51 DMSO
2.50 DMSO
2.50 DMSO
2.50 DMSO
2.40
2.40
2.35
2.34
0.00
1.23
2.12
0.80
3.14
4.83
4.87
12.0
11.5
11.0
10.5
10.0
9.5
9.0
8.5
8.0
7.5
7.0
6.5
6.0
5.5
5.0
4.5
4.0
3.5
3.0
2.5
2.0
1.5
1.0
0.5
0.0
f1 (ppm)

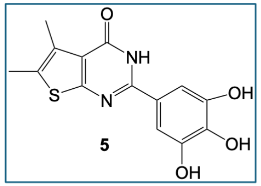
O
NH
S
N
OH
OH
OH
5

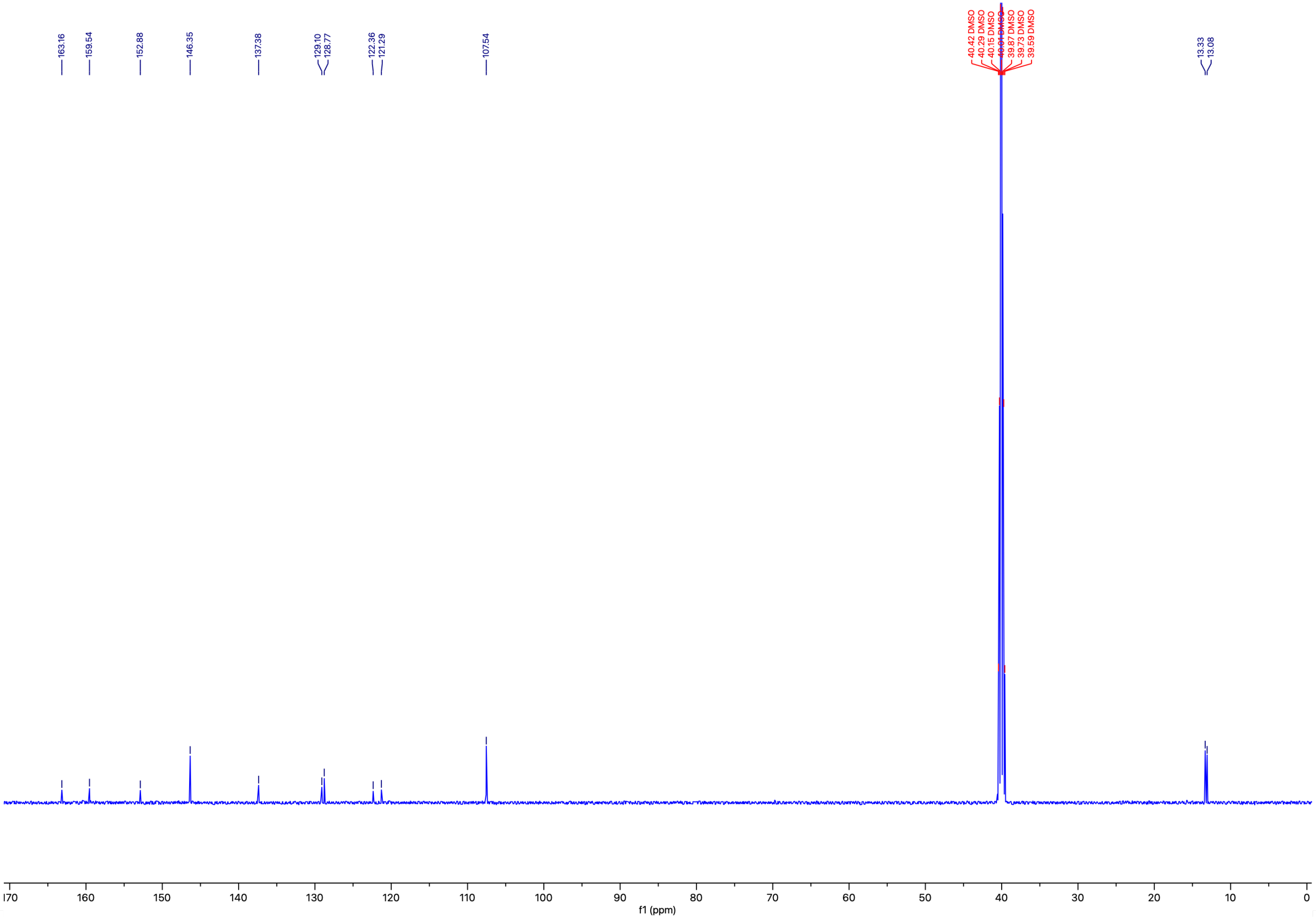
163.16
159.54
152.88
146.35
137.38
129.10
128.77
122.36
121.29
107.54
40.42 DMSO
40.29 DMSO
40.15 DMSO
40.01 DMSO
39.87 DMSO
39.73 DMSO
39.59 DMSO
13.33
13.08
170 160 150 140 130 120 110 100 90 80 70 60 50 40 30 20 10 0
f1 (ppm)

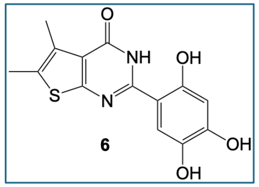

6

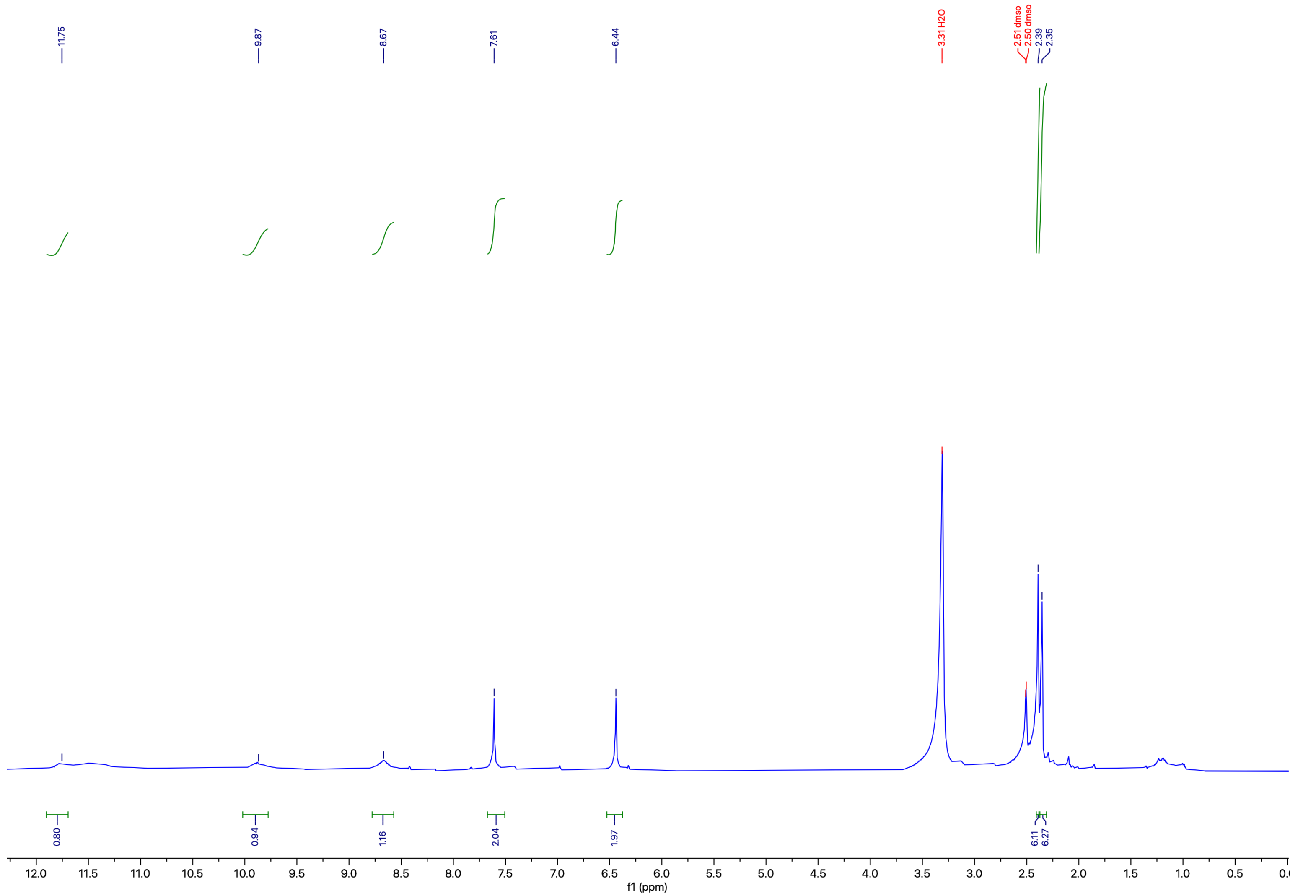
11.75
9.87
8.67
7.61
6.44
3.31 H2O
2.51 dmso
2.50 dmso
2.39
2.35
0.80
0.94
1.16
2.04
1.97
6.11
6.27
12.0 11.5 11.0 10.5 10.0 9.5 9.0 8.5 8.0 7.5 7.0 6.5 6.0 5.5 5.0 4.5 4.0 3.5 3.0 2.5 2.0 1.5 1.0 0.5 0.0
f1 (ppm)

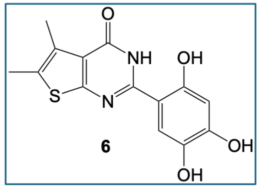
O
NH
OH
S
N
OH
6
OH

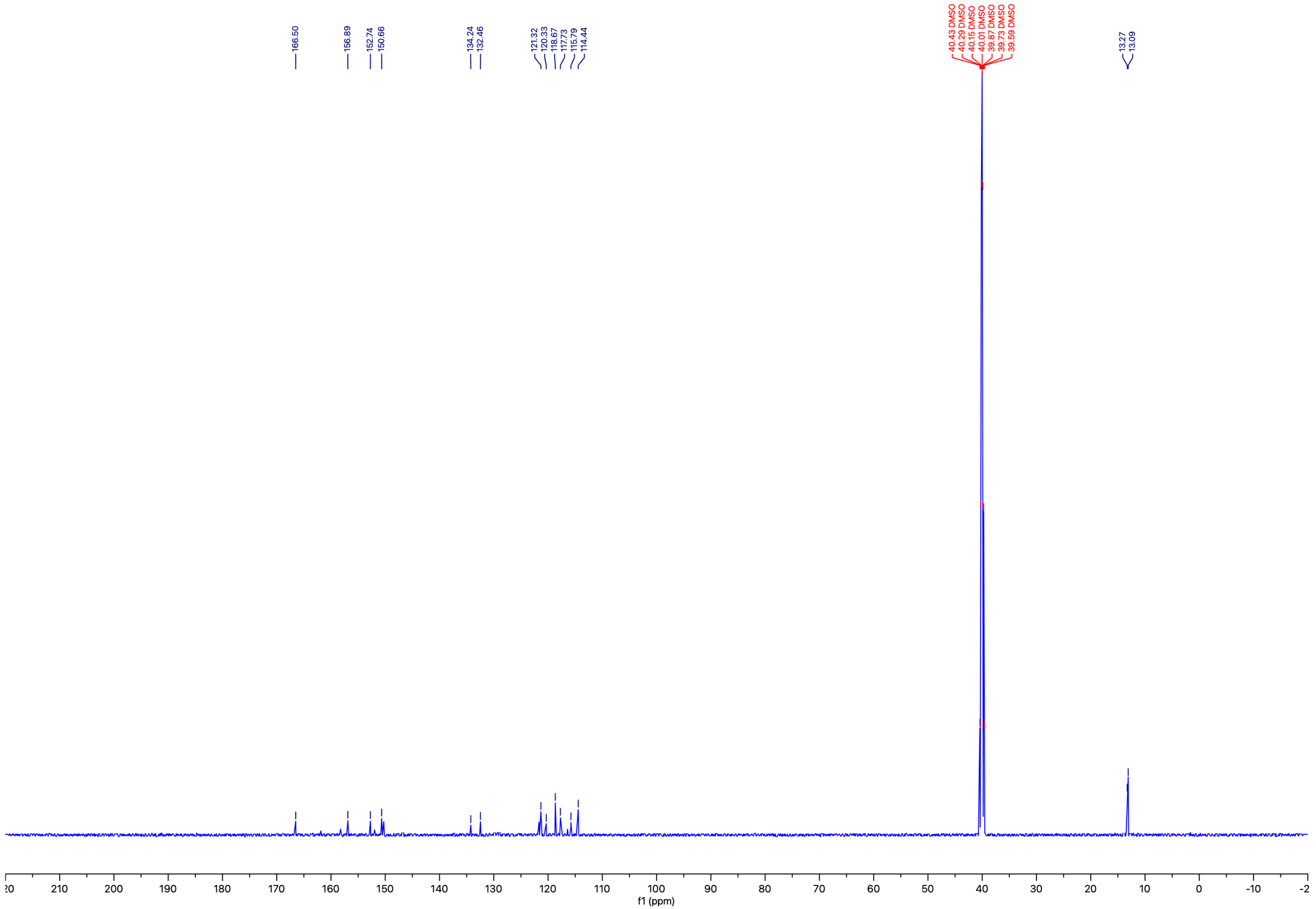
166.50
156.89
152.74
150.66
134.24
132.46
121.32
120.33
118.67
117.73
115.79
114.44
40.43 DMSO
40.29 DMSO
40.15 DMSO
40.01 DMSO
39.87 DMSO
39.73 DMSO
39.59 DMSO
13.27
13.09
f1 (ppm)

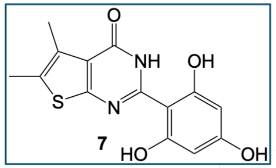
O
NH
OH
S
N
HO
OH
7

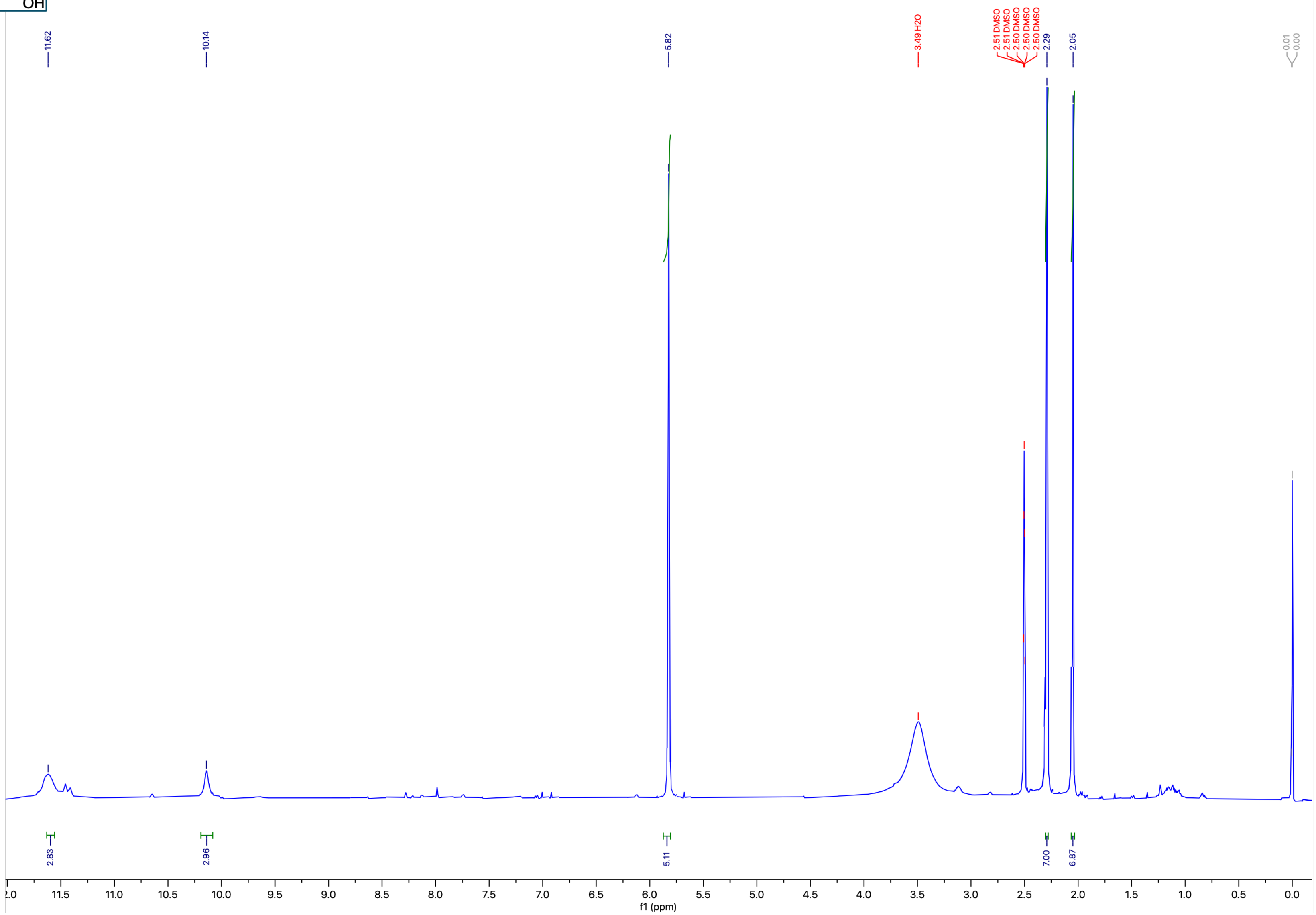
11.62
10.14
5.82
3.49 H2O
2.51 DMSO
2.51 DMSO
2.50 DMSO
2.50 DMSO
2.50 DMSO
2.29
2.05
0.01
0.00
2.83
2.96
5.11
7.00
6.87
f1 (ppm)

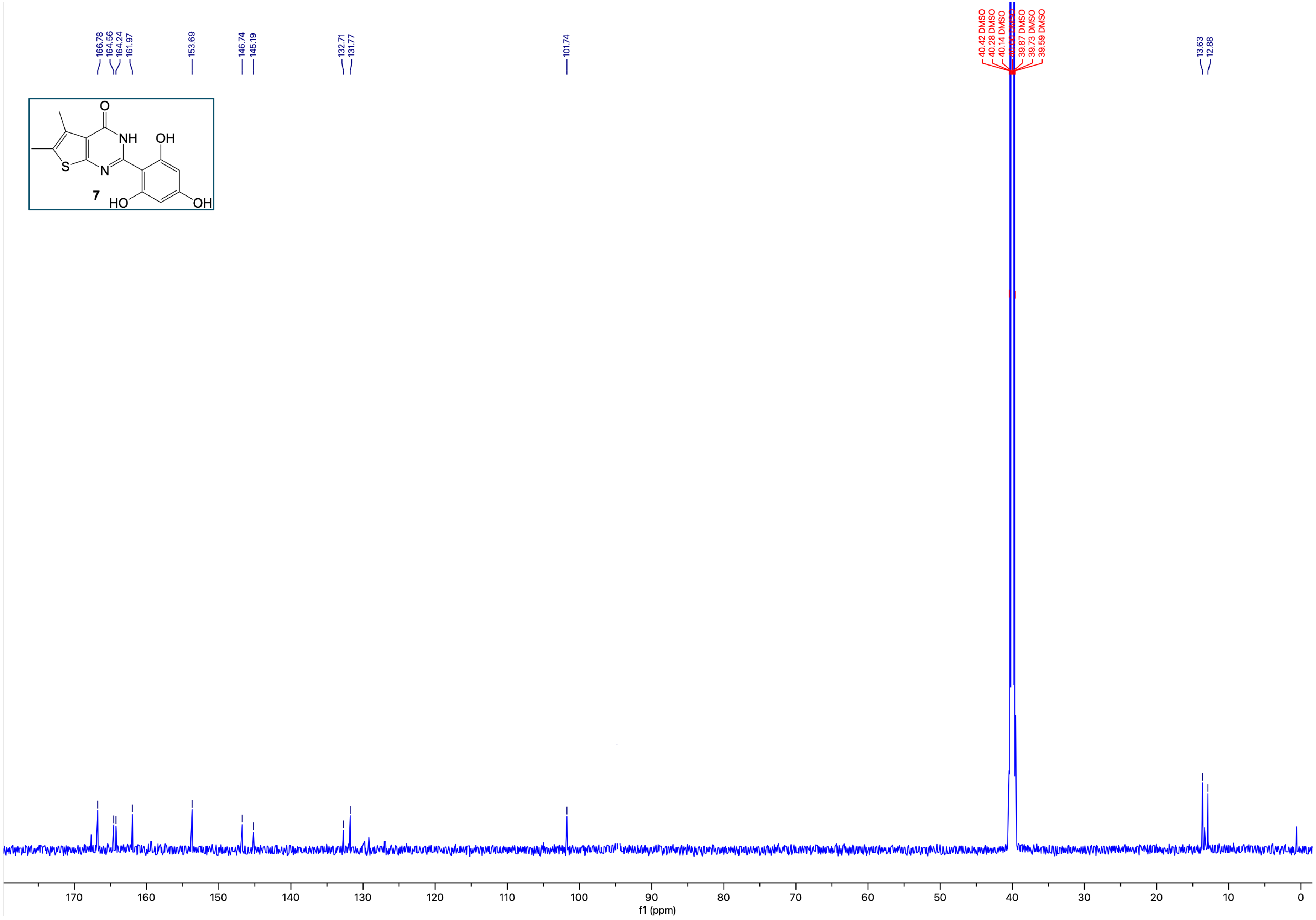

166.78
164.56
164.24
161.97
153.69
146.74
145.19
132.71
131.77
101.74
40.42 DMSO
40.28 DMSO
40.14 DMSO
39.87 DMSO
39.73 DMSO
39.59 DMSO
13.63
12.88
O
NH
OH
S
N
7
HO
OH
f1 (ppm)

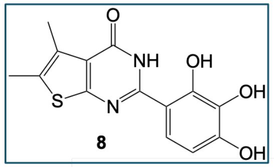
O
NH
OH
OH
S
N
OH
8

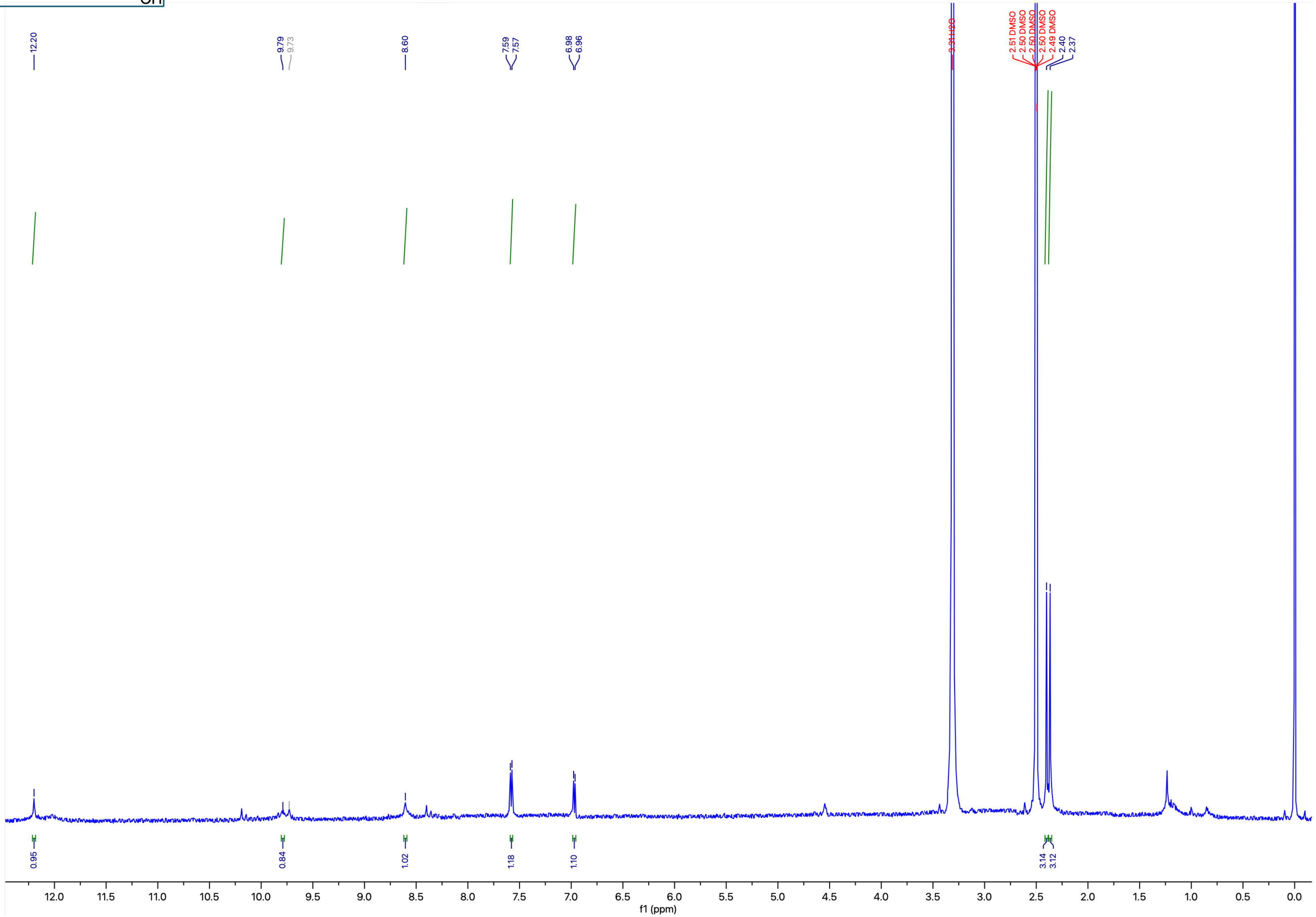
12.20
9.79
9.73
8.60
7.59
7.57
6.98
6.96
3.31 H2O
2.51 DMSO
2.50 DMSO
2.50 DMSO
2.50 DMSO
2.49 DMSO
2.40
2.37
0.95
0.84
1.02
1.18
1.10
3.14
3.12
12.0
11.5
11.0
10.5
10.0
9.5
9.0
8.5
8.0
7.5
7.0
6.5
6.0
5.5
5.0
4.5
4.0
3.5
3.0
2.5
2.0
1.5
1.0
0.5
0.0
f1 (ppm)

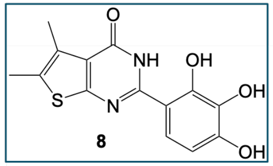
O
NH
OH
OH
S
N
OH

8

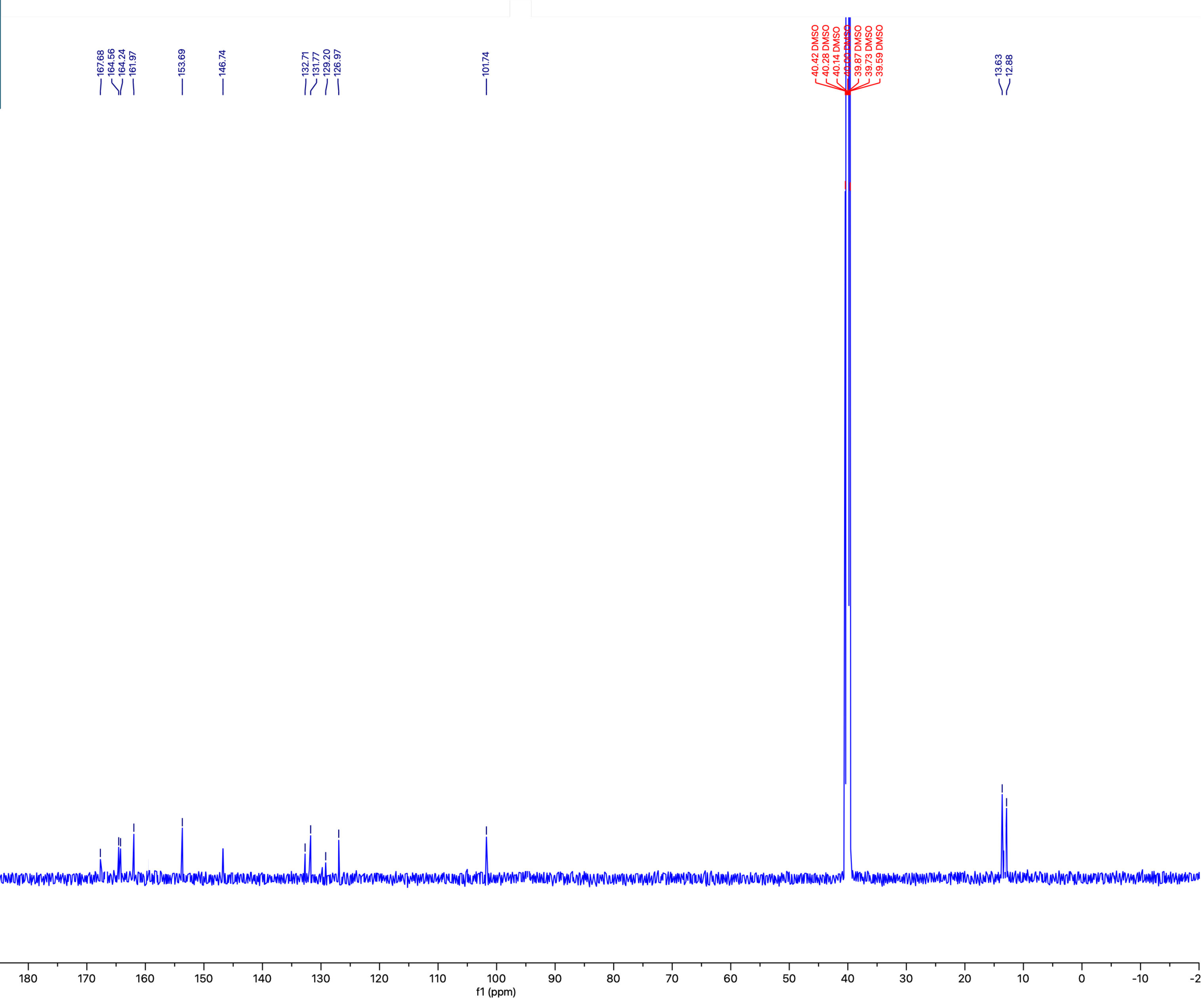
167.68
164.56
164.24
161.97
153.69
146.74
132.71
131.77
129.20
126.97
101.74
40.42 DMSO
40.28 DMSO
40.14 DMSO
39.87 DMSO
39.73 DMSO
39.59 DMSO
13.63
12.88
210
200
190
180
170
160
150
140
130
120
110
100
90
80
70
60
50
40
30
20
10
0
-10
f1 (ppm)

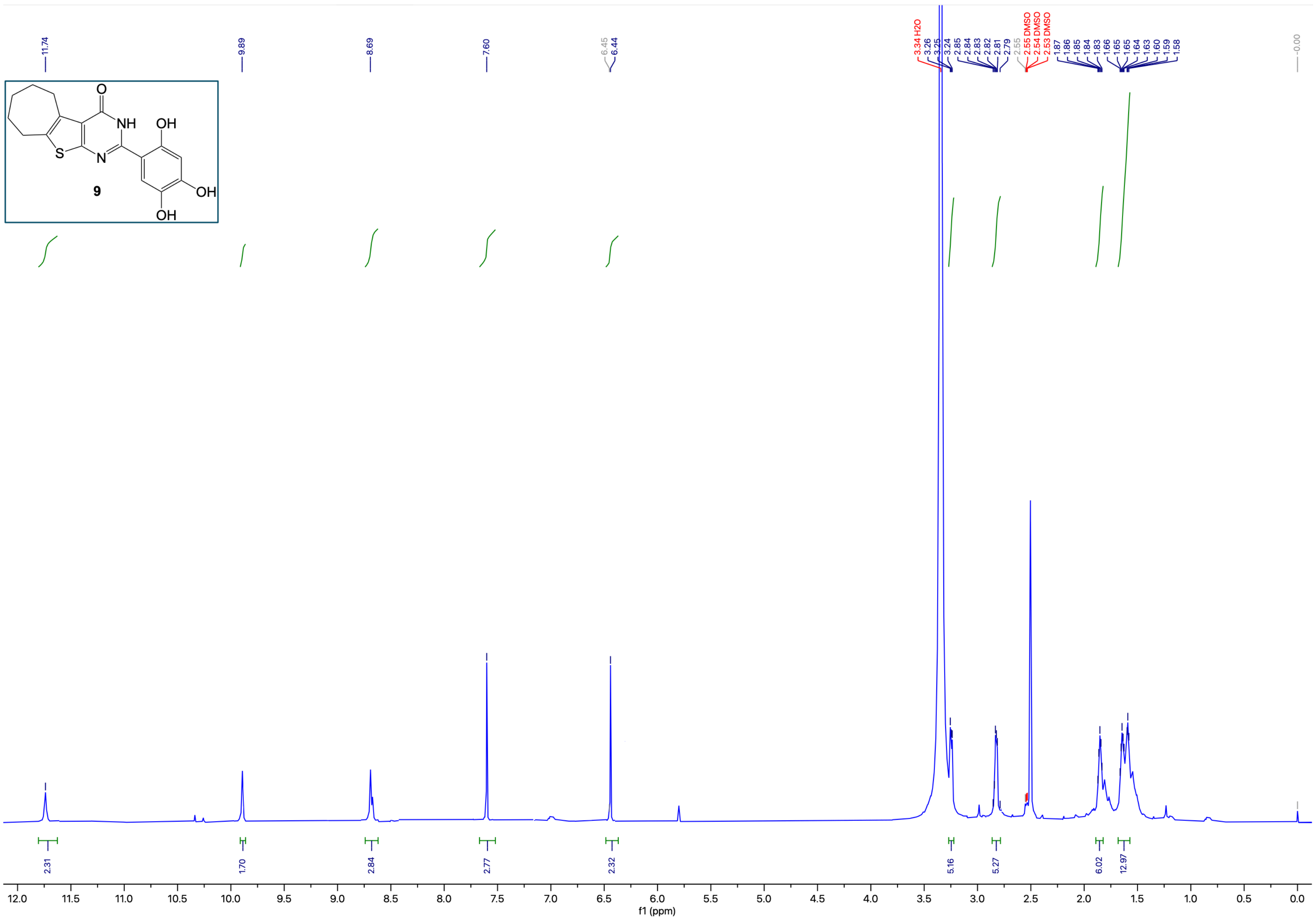
9
11.74
9.89
8.69
7.60
6.45
6.44
3.34 H2O
2.55 DMSO
2.54 DMSO
2.53 DMSO
0.00
2.31
1.70
2.84
2.77
2.32
5.16
5.27
6.02
12.97
f1 (ppm)

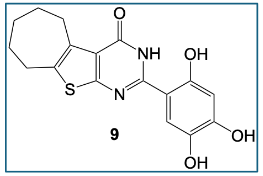
O
NH
OH
S
N
OH
OH

9

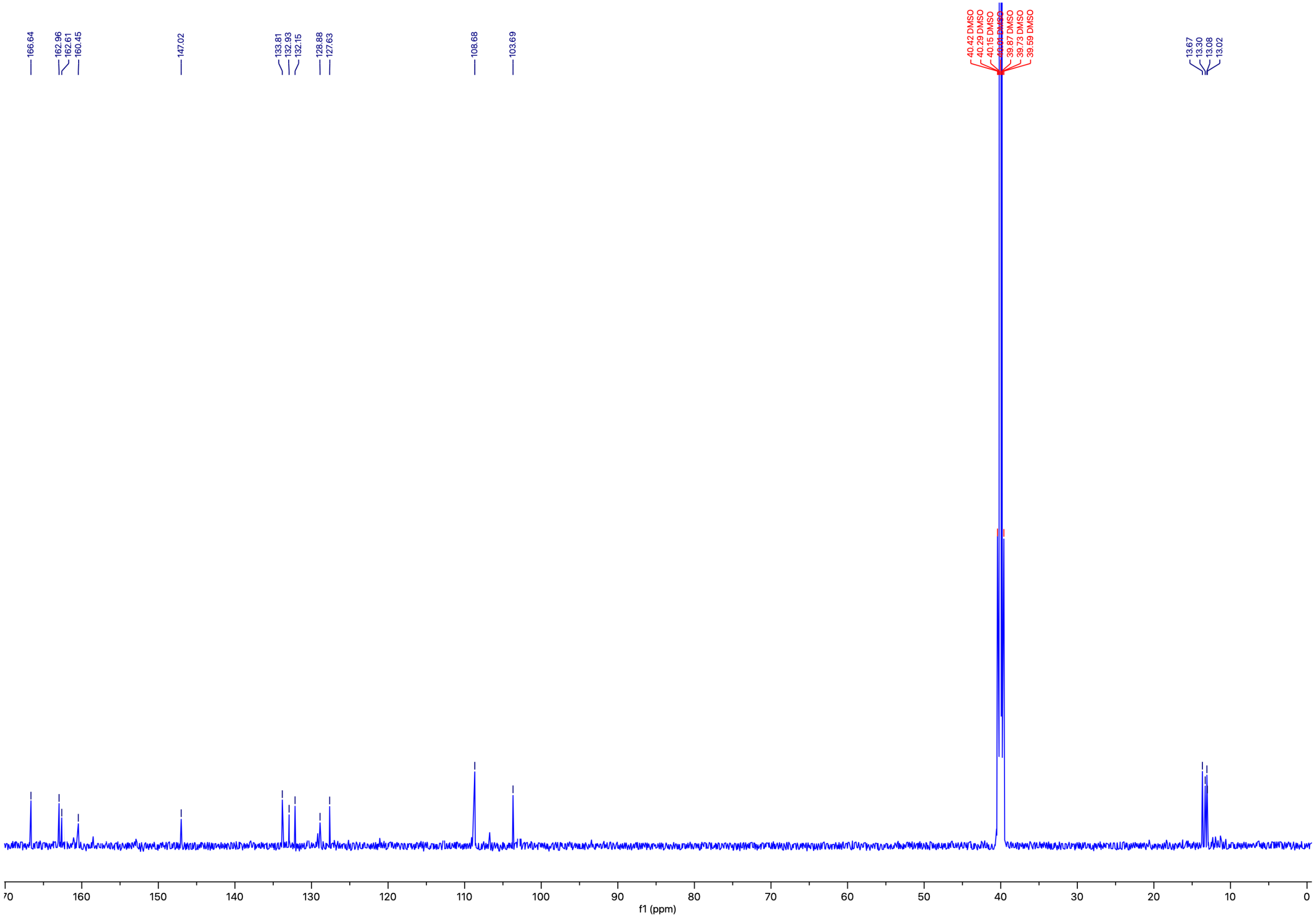
166.64
162.96
162.61
160.45
147.02
133.81
132.93
132.15
128.88
127.63
108.68
103.69
40.42 DMSO
40.29 DMSO
40.15 DMSO
40.01 DMSO
39.87 DMSO
39.73 DMSO
39.59 DMSO
13.67
13.30
13.08
13.02
70
160
150
140
130
120
110
100
90
80
70
60
50
40
30
20
10
0
f1 (ppm)

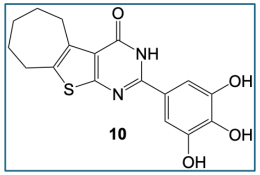
O
NH
OH
S
N
OH
10
OH

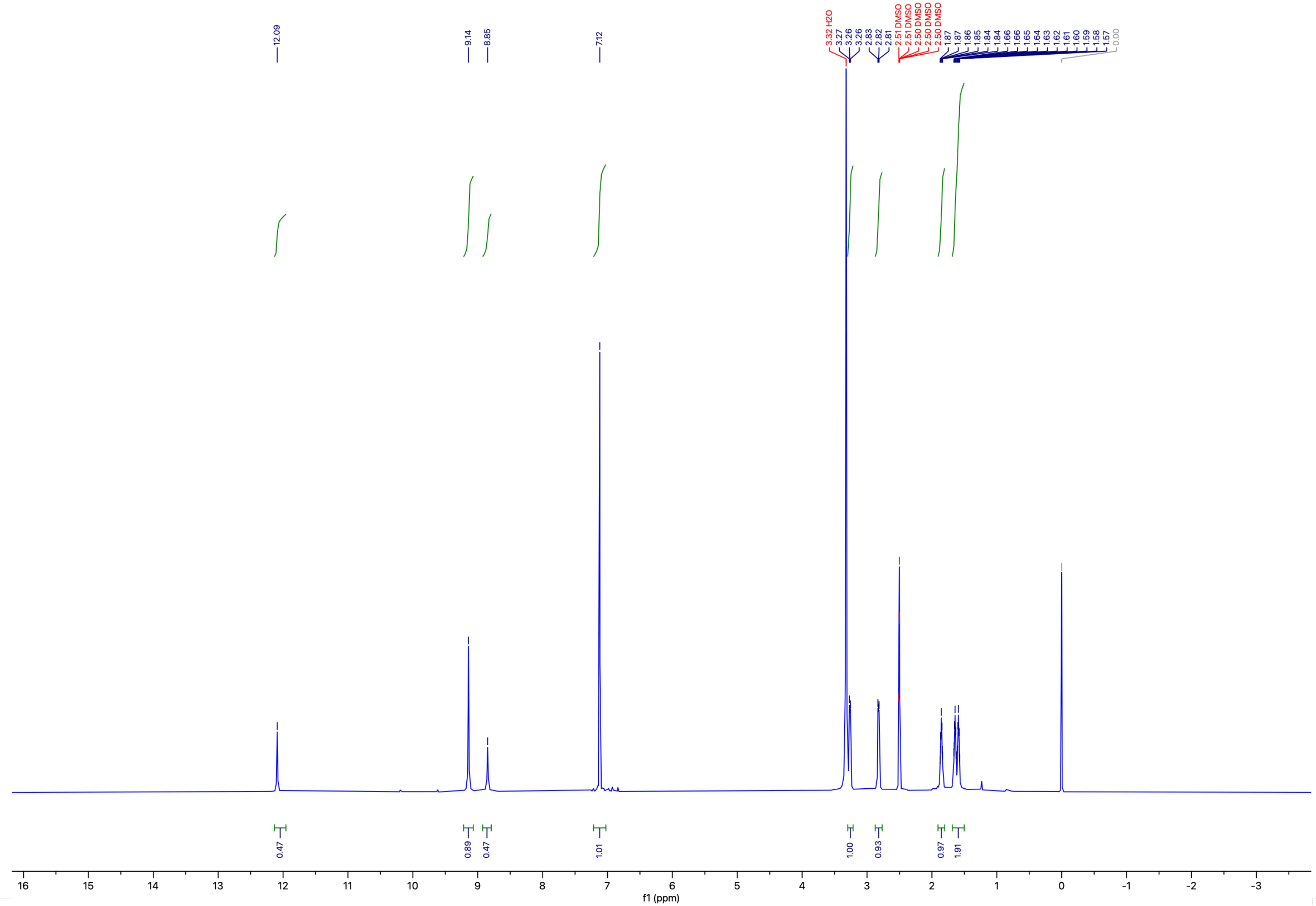
12.09
9.14
8.85
7.12
3.32 H2O
3.27
3.26
3.26
2.83
2.82
2.81
2.51 DMSO
2.51 DMSO
2.50 DMSO
2.50 DMSO
2.50 DMSO
1.87
1.87
1.86
1.85
1.84
1.66
1.66
1.65
1.64
1.63
1.62
1.61
1.60
1.59
1.58
1.57
0.00
0.47
0.89
0.47
1.01
1.00
0.93
0.97
1.91
16 15 14 13 12 11 10 9 8 7 6 5 4 3 2 1 0 -1 -2 -3
f1 (ppm)

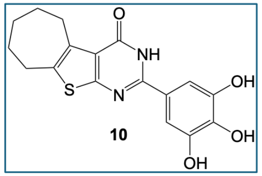
O
NH
N
S
OH
OH
OH
10

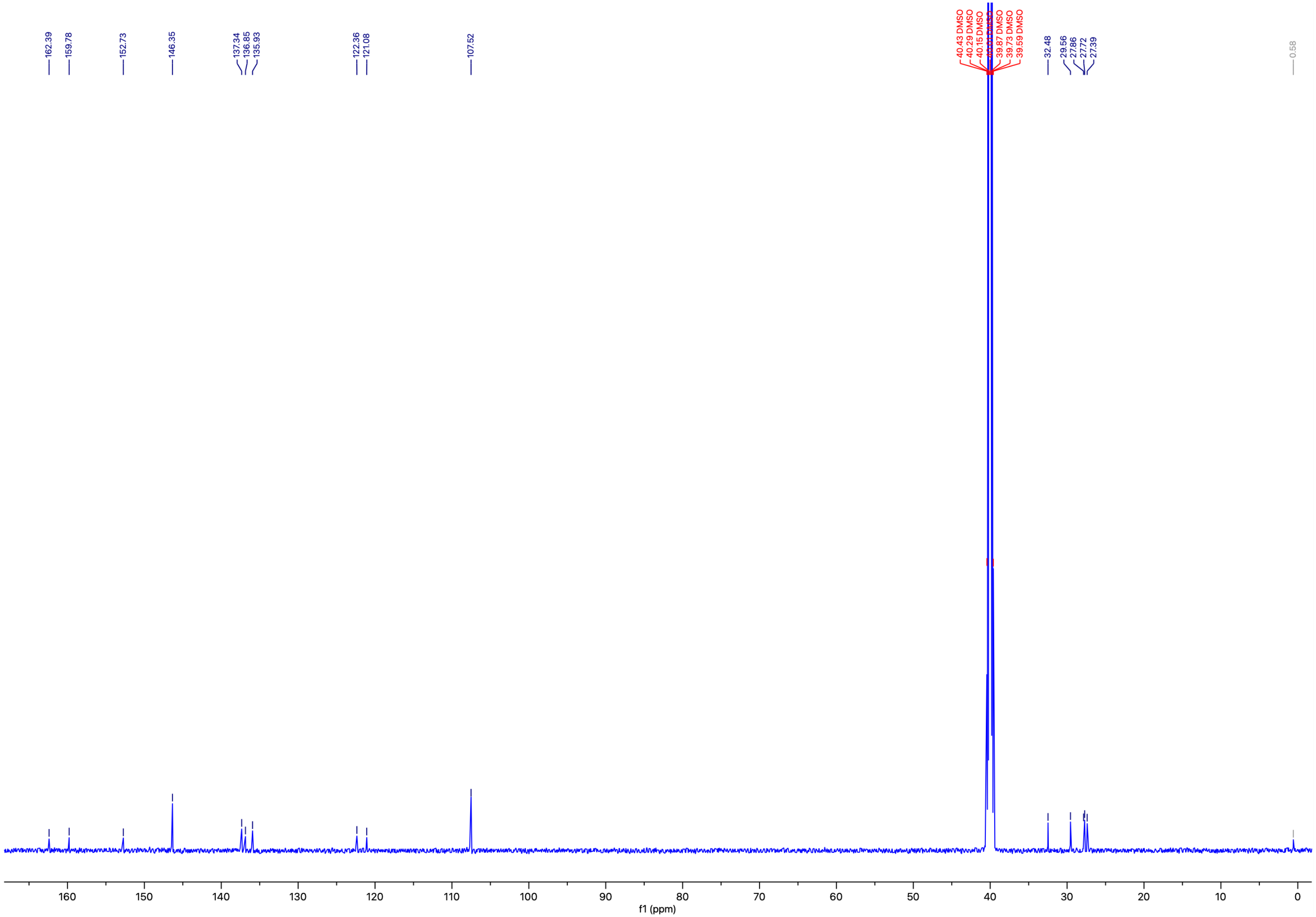
162.39
159.78
152.73
146.35
137.34
136.85
135.93
122.36
121.08
107.52
40.43 DMSO
40.29 DMSO
40.15 DMSO
39.87 DMSO
39.73 DMSO
39.59 DMSO
32.48
29.56
27.86
27.72
27.39
0.58
160
150
140
130
120
110
100
90
80
70
60
50
40
30
20
10
0
f1 (ppm)

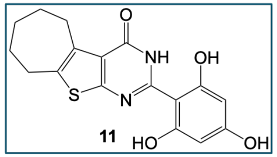
O
NH
OH
S
N
11
HO
OH

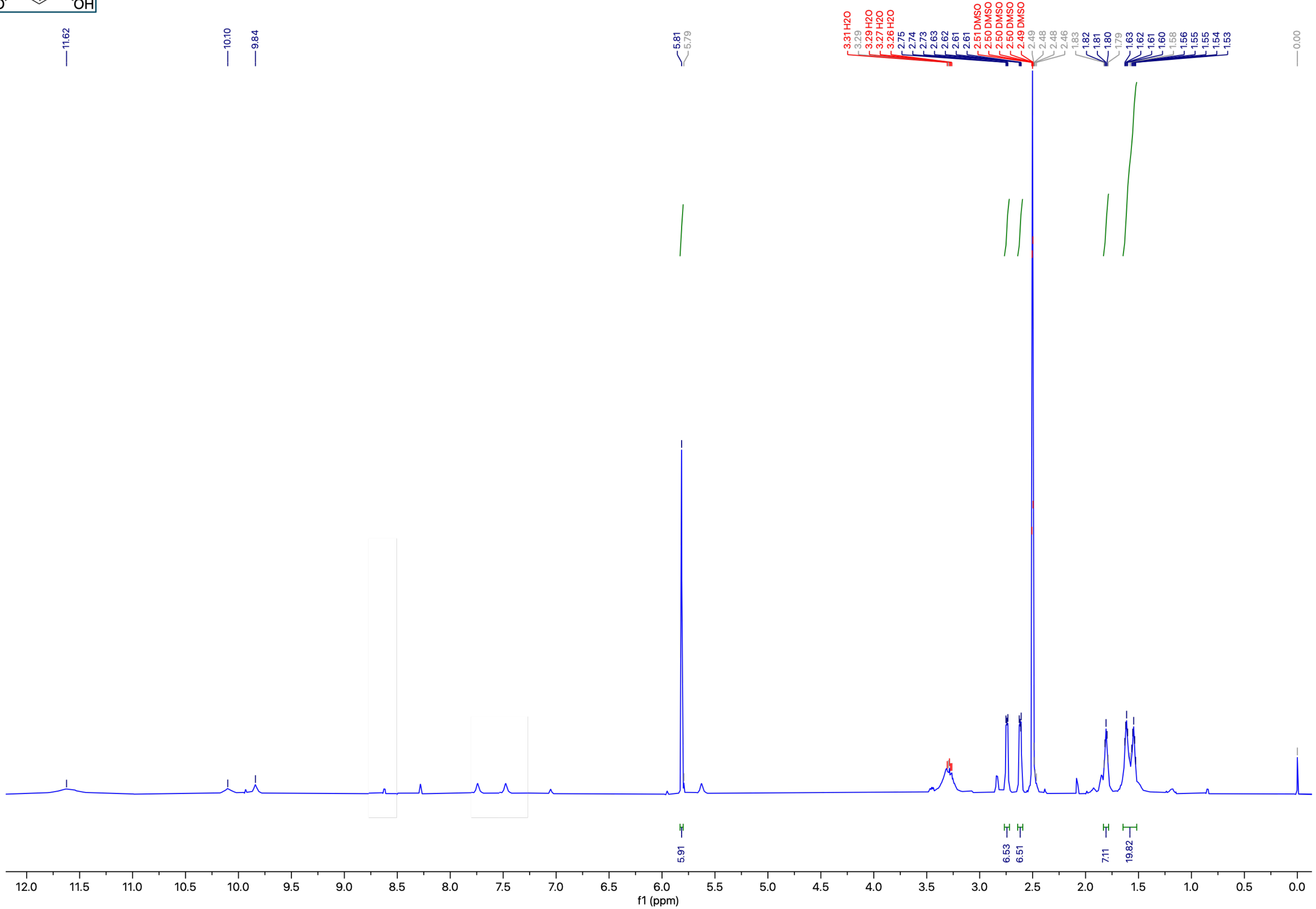
11.62
10.10
9.84
5.81
5.79
3.31 H2O
3.29
3.29 H2O
3.27 H2O
3.26 H2O
2.75
2.74
2.73
2.63
2.62
2.61
2.61
2.51 DMSO
2.50 DMSO
2.50 DMSO
2.50 DMSO
2.49 DMSO
2.49
2.48
2.48
2.46
1.83
1.82
1.81
1.80
1.79
1.63
1.62
1.61
1.60
1.58
1.56
1.55
1.55
1.54
1.53
0.00
5.91
6.53
6.51
7.11
19.82
12.0
11.5
11.0
10.5
10.0
9.5
9.0
8.5
8.0
7.5
7.0
6.5
6.0
5.5
5.0
4.5
4.0
3.5
3.0
2.5
2.0
1.5
1.0
0.5
0.0
f1 (ppm)

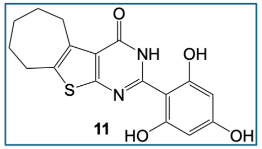
O
NH
OH
S
N
HO
OH

11

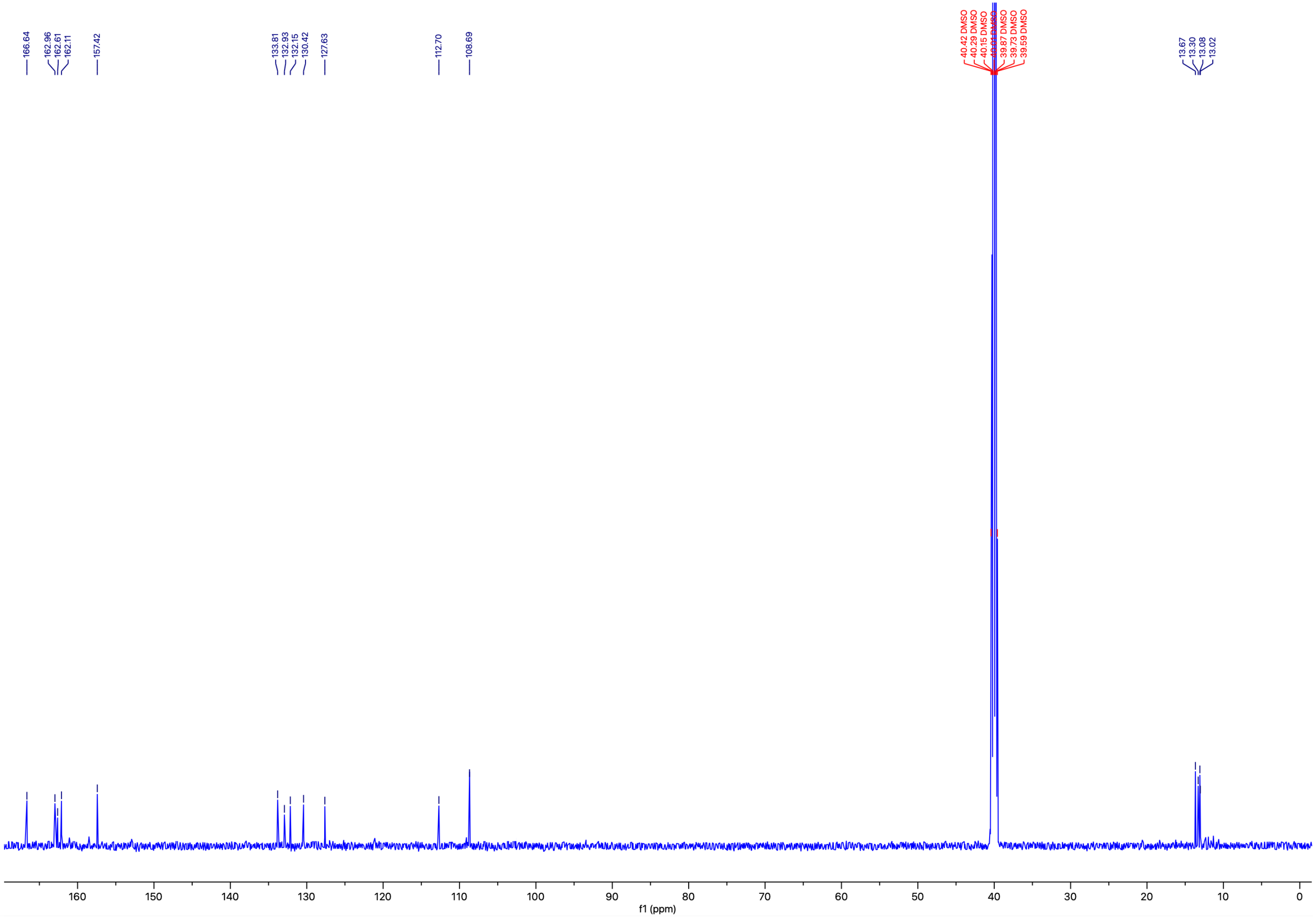
166.64
162.96
162.61
162.11
157.42
133.81
132.93
132.15
130.42
127.63
112.70
108.69
40.42 DMSO
40.29 DMSO
40.15 DMSO
39.87 DMSO
39.73 DMSO
39.59 DMSO
13.67
13.30
13.08
13.02
160
150
140
130
120
110
100
90
80
70
60
50
40
30
20
10
0
f1 (ppm)

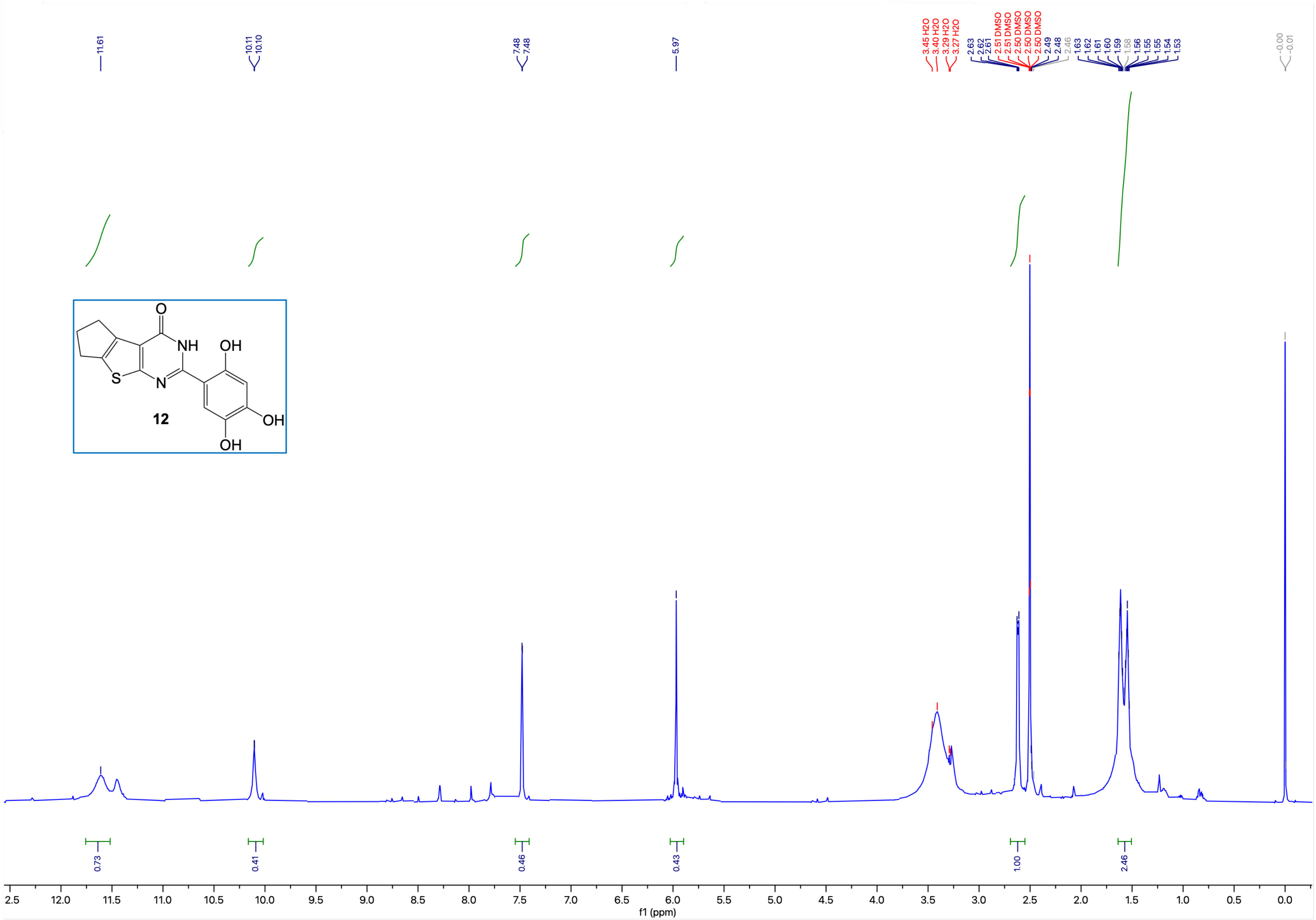
11.61
10.11
10.10
7.48
7.48
5.97
3.45 H2O
3.40 H2O
3.29 H2O
3.27 H2O
2.63
2.62
2.61
2.51 DMSO
2.51 DMSO
2.50 DMSO
2.50 DMSO
2.50 DMSO
2.49
2.48
2.46
1.63
1.62
1.61
1.60
1.59
1.58
1.56
1.55
1.55
1.54
1.53
0.00
-0.01
O
NH
OH
S
N
12
OH
OH
0.73
0.41
0.46
0.43
1.00
2.46
f1 (ppm)

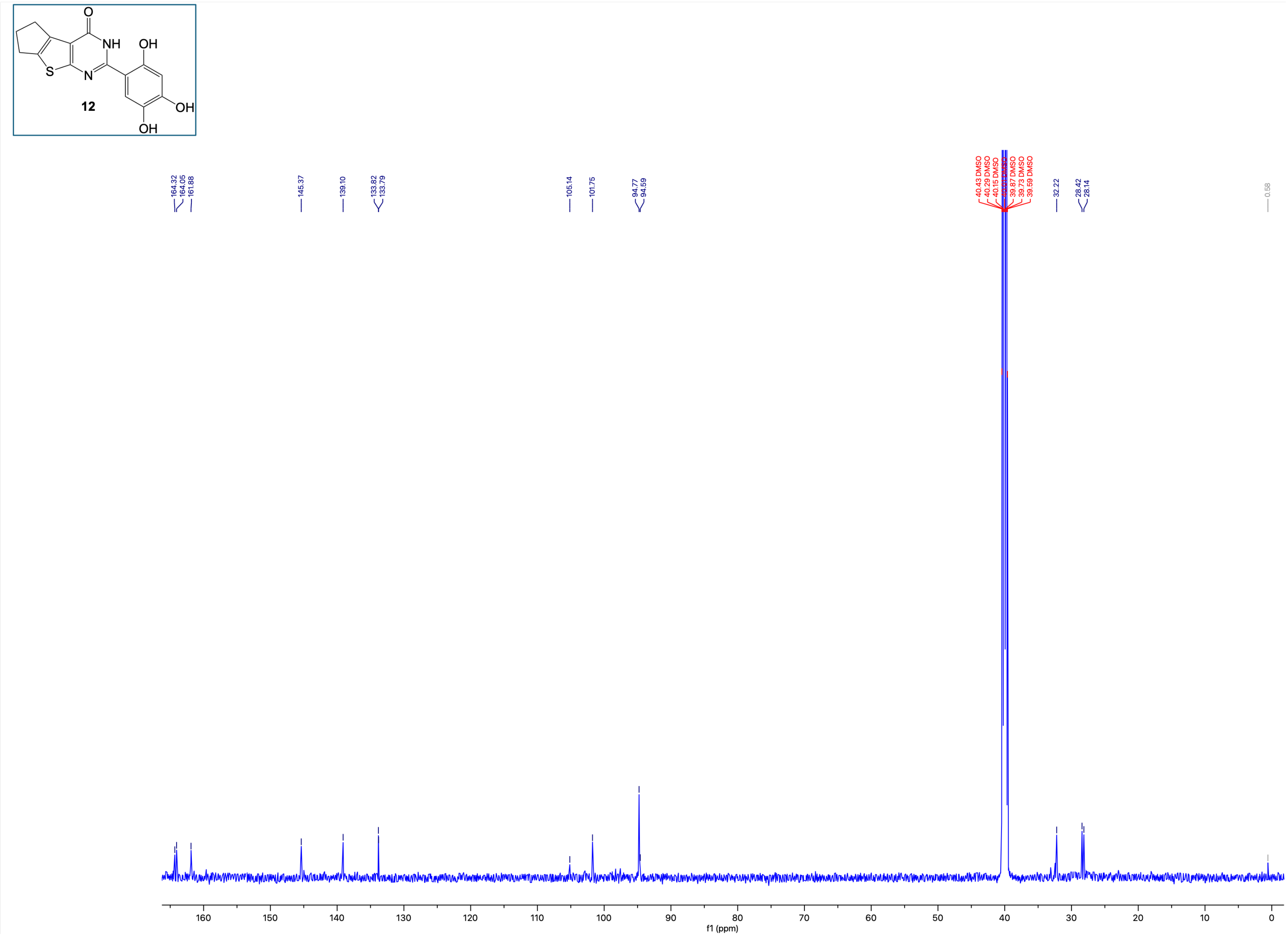

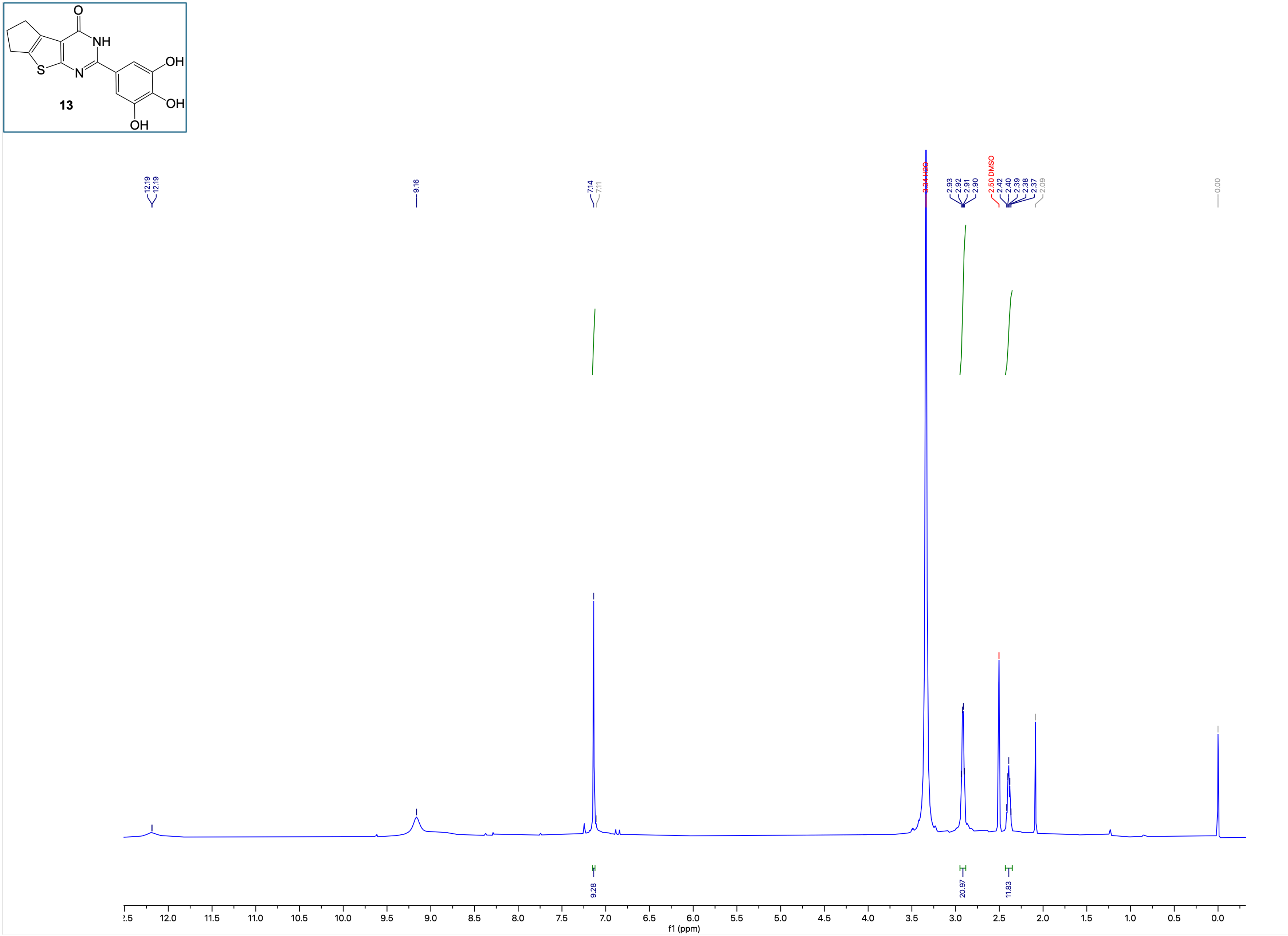
O
NH
OH
OH
OH
S
N
13
12.19
12.19
9.16
7.14
7.11
3.34 H2O
2.93
2.92
2.91
2.90
2.50 DMSO
2.42
2.40
2.39
2.38
2.37
2.09
0.00
9.28
20.97
11.83
12.0 11.5 11.0 10.5 10.0 9.5 9.0 8.5 8.0 7.5 7.0 6.5 6.0 5.5 5.0 4.5 4.0 3.5 3.0 2.5 2.0 1.5 1.0 0.5 0.0
f1 (ppm)

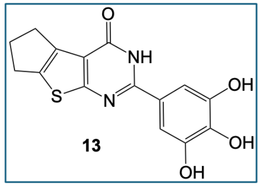
O
NH
OH
S
N
OH
OH

13

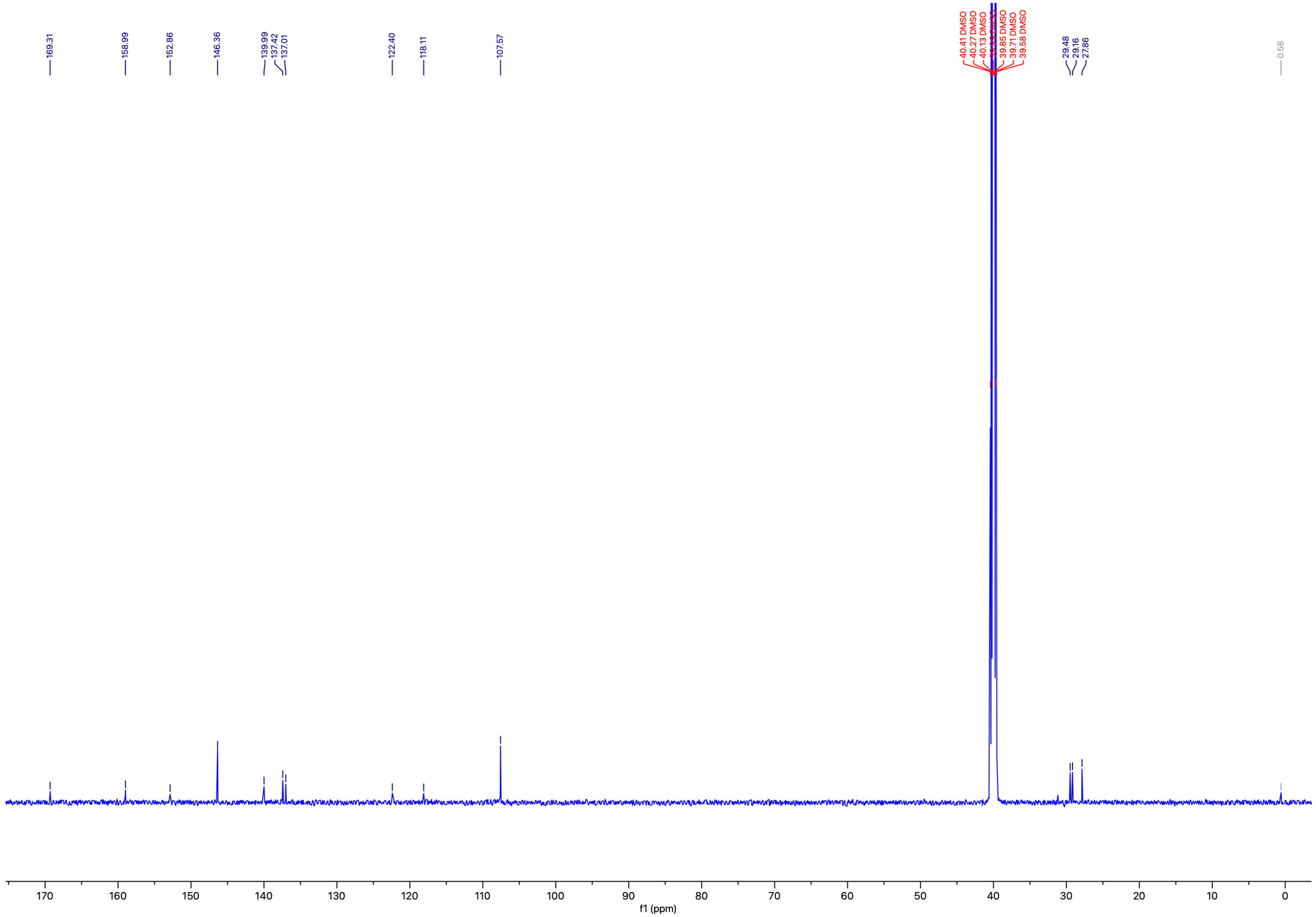
169.31
158.99
152.86
146.36
139.99
137.42
137.01
122.40
118.11
107.57
40.41 DMSO
40.27 DMSO
40.13 DMSO
39.85 DMSO
39.71 DMSO
39.58 DMSO
29.48
29.16
27.86
0.58
170
160
150
140
130
120
110
100
90
80
70
60
50
40
30
20
10
0
f1 (ppm)

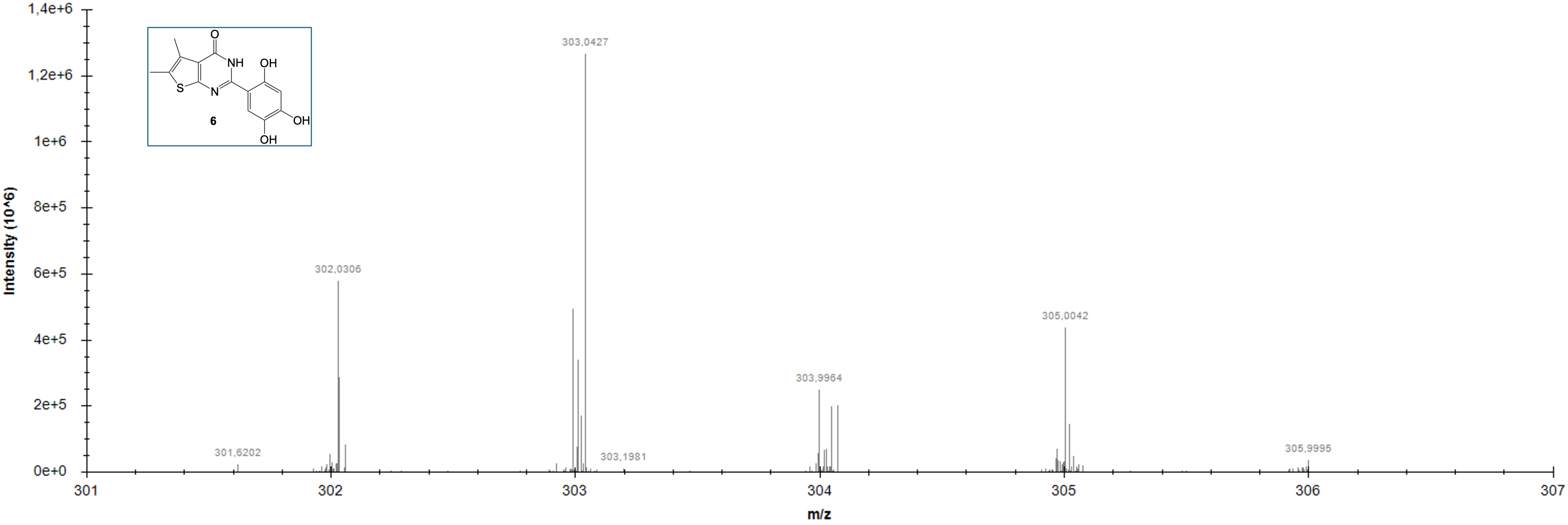

1,4e+6
1,2e+6
1e+6
8e+5
6e+5
4e+5
2e+5
0e+0
Intensity (10^6)
O
NH
OH
S
N
OH
OH
6
303,0427
302,0306
305,0042
303,9964
301,6202
303,1981
305,9995
301
302
303
304
305
306
307
m/z

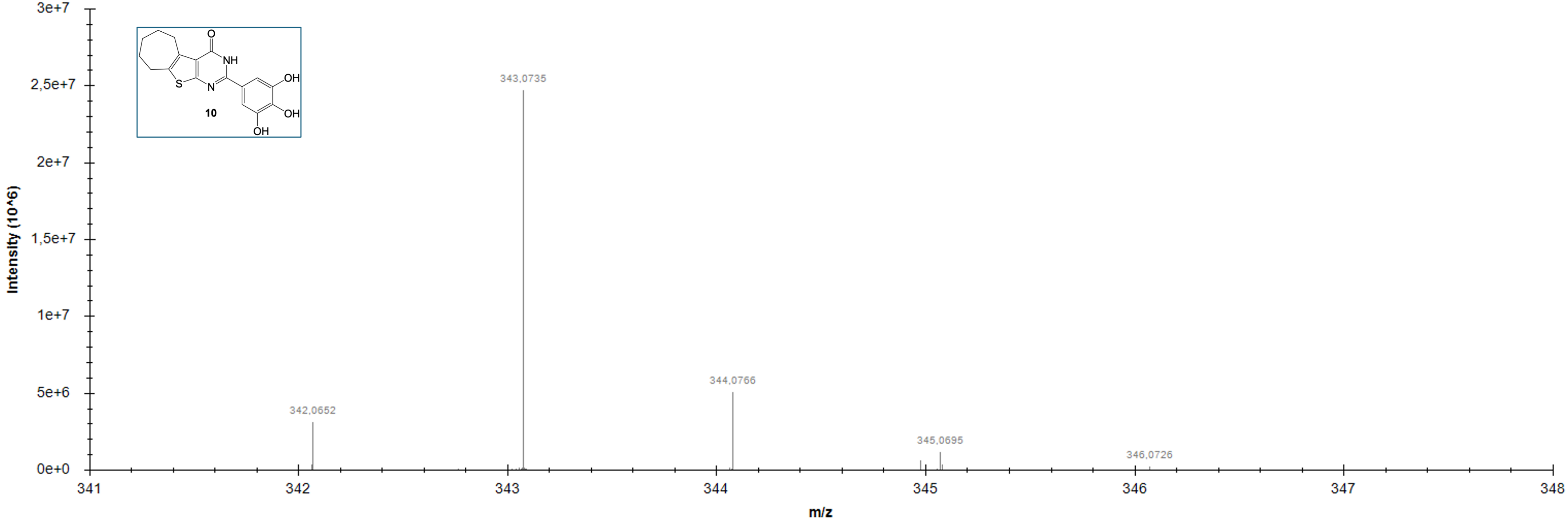

O
NH
S
N
OH
OH
OH
10
3e+7
2,5e+7
2e+7
1,5e+7
1e+7
5e+6
0e+0
Intensity (10^6)
343,0735
344,0766
342,0652
345,0695
346,0726
341
342
343
344
345
346
347
348
m/z

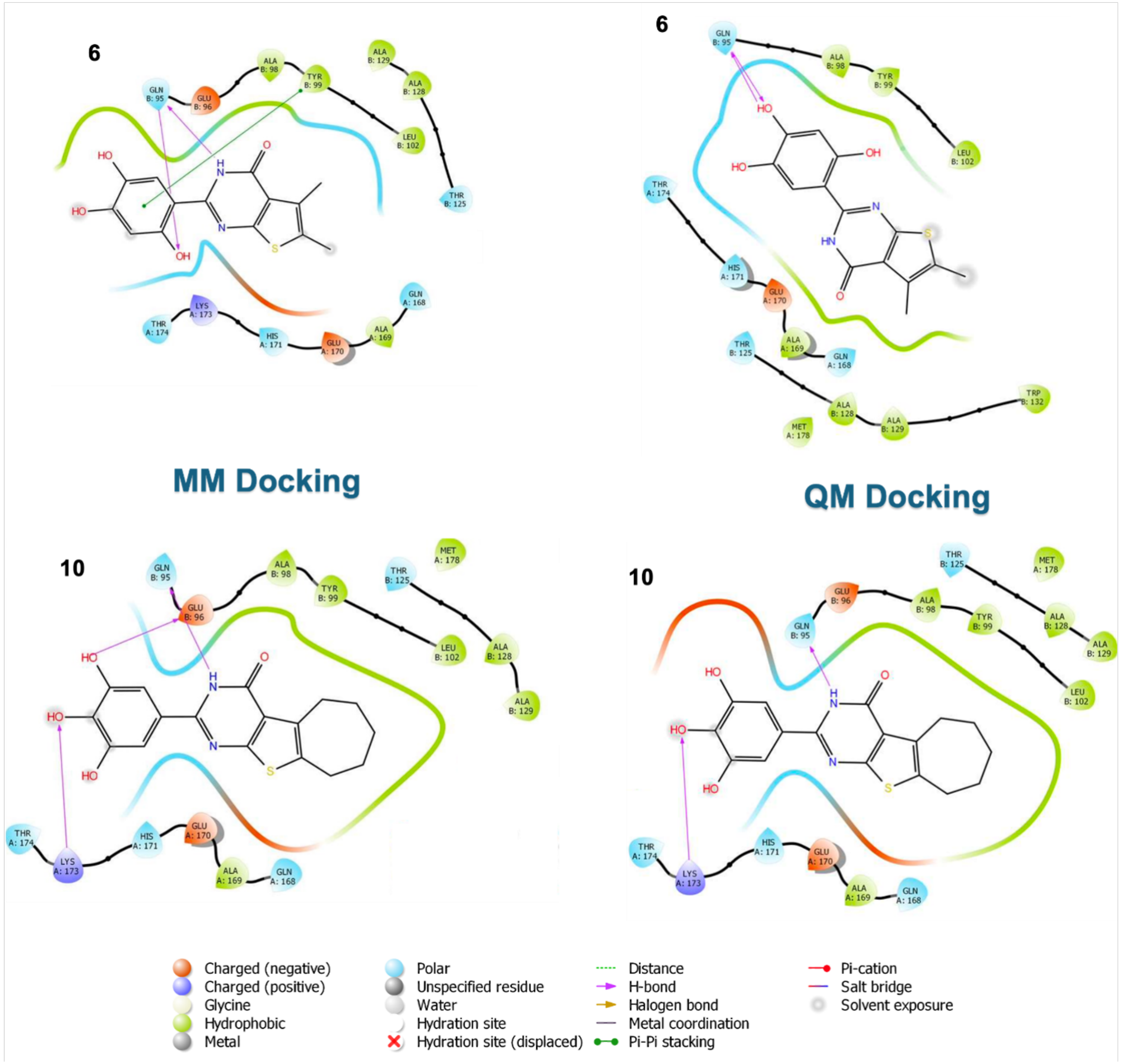

Figure S1. Interactions of compounds 6 and 10 with the region between the SBS and the LEDGINs BS (**6** and **10** BS) in the presence of sucrose identified by MM and QM approaches

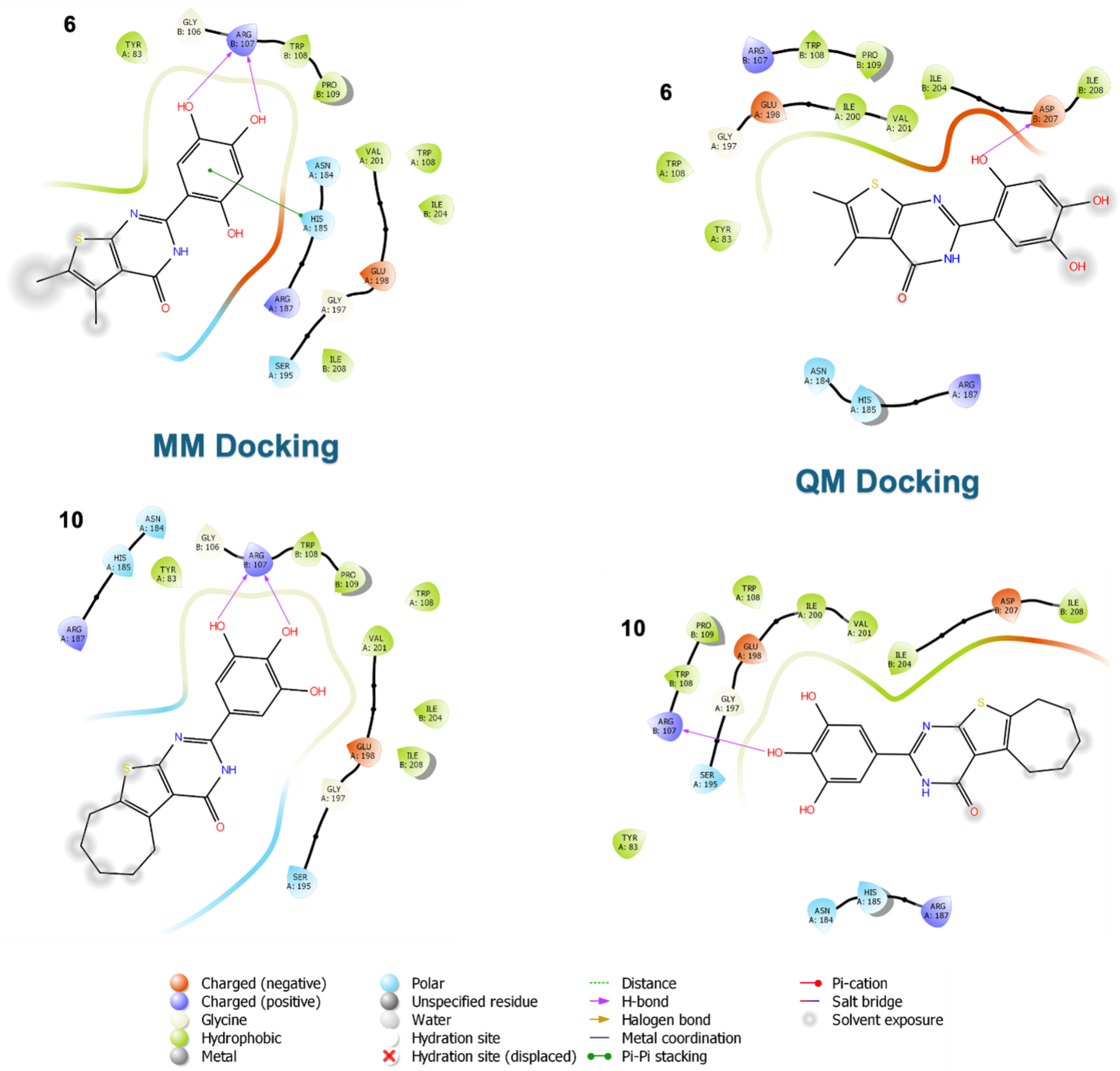

Figure S2. Interactions of compounds **6** and **10** and the Unexplored BS, identified by MM and QM approaches

Pocket in between the SBS and the LEDGINs BS (6 and 10 BS) (in the absence of sucrose)

Compound 6

Aminoacid	VdW (Kcal/mol)	Coulomb (Kcal/mol)	H-bond (Kcal/mol)	Distance (Å)	Internal Energy (Kcal/mol)
Lys 173	-3.090	-3.222	0.00	2.712	-6.312
Glu 96	-2.481	-15.595	-0.500	1.783	-18.576
Gln 95	-5.732	-2.762	-0.748	2.139	-9.242
Tyr 99	-0.819	1.932	0.00	1.721	1.113

Compound 10

Aminoacid	VdW (Kcal/mol)	Coulomb (Kcal/mol)	H-bond (Kcal/mol)	Distance (Å)	Internal Energy (Kcal/mol)
Lys 173	-3.421	-9.018	-0.500	2.061	-12.939
Glu 96	-0.840	-10.731	-0.537	1.876	-12.108
Gln 95	-5.485	-0.703	-0.041	2.463	-6.229
Tyr 99	-2.869	-2.073	0.00	1.567	-0.796

Figure S3. Estimation of the contributions and energy gains of all amino acids located within a 12Å radius of the ligands. [pocket in between the SBS and the LEDGINs BS (**6** and **10** BS) without sucrose)]

Pocket in between the SBS and the LEDGINs BS (6 and 10BS) (in the presence of sucrose)

Compound 6

Aminoacid	VdW (Kcal/mol)	Coulomb (Kcal/mol)	H-bond (Kcal/mol)	Distance (Å)	Internal Energy (Kcal/mol)
Lys 173	-1.911	-2.583	0.00	2.475	0.672
Glu 96	-2.525	-5.100	0.00	2.907	-7.625
Gln 95	-2.737	-3.324	-0.701	1.898	-6.762
Tyr 99	-0.977	1.479	0.00	1.897	-0.502
Sucrose	1.401	-4.181	-2.00	1.748	-4.780

Compound 10

Aminoacid	VdW (Kcal/mol)	Coulomb (Kcal/mol)	H-bond (Kcal/mol)	Distance (Å)	Internal Energy (Kcal/mol)
Lys 173	-3.263	-7.721	-0.500	2.063	-11.484
Glu 96	-0.817	-6.808	-0.350	2.033	-7.974
Gln 95	-4.038	-0.764	0.00	2.311	-4.802
Tyr 99	-4.371	1.552	0.00	1.866	-2.819
Sucrose	-1.184	1.804	0.00	2.377	0.620

Figure S4. Estimation of the contributions and energy gains of all amino acids and sucrose located within a 12Å radius of the ligands. [pocket in between the SBS and the LEDGINs BS (**6** and **10** BS)]

UNEXPLORED BINDING SITE

Compound 6

Aminoacid	VdW (Kcal/mol)	Coulomb (Kcal/mol)	H-bond (Kcal/mol)	Distance (Å)	Internal Energy (Kcal/mol)
Arg 107	-0.494	-3.284	-2.00	1.950	-5.778
Trp 108	-2.983	-1.131	0.00	2.638	-4.114
Pro 109	-3,768	-0.357	0.00	2.594	-4.126
His 185	-7.123	0.999	0.00	2.782	-6.124
Arg 187	-0,816	-5.092	0.00	2.881	-5.908
Ser 195	-1.785	-2.775	-0.630	2.248	-5.190

Compound 10

Aminoacid	VdW (Kcal/mol)	Coulomb (Kcal/mol)	H-bond (Kcal/mol)	Distance (Å)	Internal Energy (Kcal/mol)
Arg 107	-1.211	-2.413	-1.886	2.001	-5.510
Trp 108	-2.669	-1.284	0.00	2.723	-3.953
Pro 109	-3.893	-0.352	0.00	2.731	-4.245
His 185	-7.398	1.201	0.00	2.727	-6.197
Arg 187	-1.288	-4.967	0.00	2.910	-6.255
Ser 195	-1.450	-3.380	-1.00	2.055	-5.829

Figure S5. Estimation of the contributions and energy gains of all amino acids located within a 12Å radius of the ligands. (Unexplored BS)
